# Highly Efficient Photothermal Icephobic/de‐Icing MOF‐Based Micro and Nanostructured Surface

**DOI:** 10.1002/advs.202304187

**Published:** 2023-08-26

**Authors:** Lei Zhang, Bingcai Luo, Kun Fu, Chunlei Gao, Xuefeng Han, Maolin Zhou, Tiance Zhang, Lieshuang Zhong, Yongping Hou, Yongmei Zheng

**Affiliations:** ^1^ Key Laboratory of Bio‐Inspired Smart Interfacial Science and Technology of Ministry of Education School of Chemistry Beihang University (BUAA) Beijing 100191 P. R. China; ^2^ Key Laboratory of Green Chemistry and Technology of Ministry of Education College of Chemistry Sichuan University 29 Wangjiang Road Chengdu 610064 P. R. China; ^3^ Key Laboratory of Yangtze River Water Environment Ministry of Education Shanghai Institute of Pollution Control and Ecological Security College of Environmental Science and Engineering Tongji University Shanghai 20092 P. R. China

**Keywords:** de‐icing, icing delay, micro‐ and nanostructure, MOF, photothermal‐conversion mechanism

## Abstract

Photothermal materials have gained considerable attention in the field of anti‐/de‐icing due to its environmental friendliness and energy saving. However, it is always significantly challenging to obtain solar thermal materials with hierarchical structure and simultaneously demonstrate both the ultra‐long icing delay ability and the superior photothermal de‐icing ability. Here, a photothermal icephobic MOF‐based micro and nanostructure surface (MOF‐MNS) is presented, which consists of micron groove structure and fluorinated MOF nanowhiskers. The optimal MOF‐M_250_NS can achieve solar absorption of over 98% and produce a high temperature increment of 65.5 °C under 1‐sun illumination. Such superior photothermal‐conversion mechanism of MOF‐M_250_NS is elucidated in depth. In addition, the MOF‐M_250_NS generates an ultra‐long icing delay time of ≈3960 s at −18 °C without solar illumination, achieving the longest delay time, which isn't reported before. Due to its excellent solar‐to‐heat conversation ability, accumulated ice and frost on MOF‐M_250_NS can be rapidly melted within 720 s under 1‐sun illumination and it also holds a high de‐icing rate of 5.8 kg m^−2^ h^−1^. MOF‐M_250_NS possesses the versatility of mechanical robustness, chemical stability, and low temperature self‐cleaning, which can synergistically reinforce the usage of icephobic surfaces in harsh conditions.

## Graphical abstract

A novel anti‐/de‐icing material (MOF‐M_250_NS) is designed by combining micron grooves and Cu‐MOF nanowires structures, achieving robust icing delay time of 3960 s at −18 °C, and high‐efficient photothermal de‐icing performance of 720 s even surface at −20 °C, due to a synergistic effect of hierarchical micro‐nanostructures for solar absorption and effective sunlight‐trapped for thermal converting.

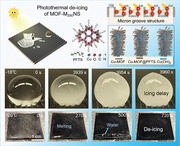

## 1 Introduction

Accumulated ice and frost on the materials can cause serious safety and economic problems.^[^ ^1^, ^2^, ^3^ ^]^ Therefore, de‐icing plays a crucial role in a wide range of industrial and commercial applications (e.g., aeroplane, power lines, ships, photovoltaic panels, vehicles, and so on).^[^ ^4^, ^5^, ^6^, ^7^, ^8^ ^]^ At present, traditional de‐icing methods, like chemical and mechanical de‐icing, suffer from low efficiency, high energy consumption, and environmental pollution.^[^ ^9^ ^]^ Fortunately, some efforts have been dedicated to photothermal anti‐/de‐icing surfaces (PADS),^[^ ^10^, ^11^, ^12^ ^]^ which can efficiently convert solar energy to heat. These surfaces utilize green and renewable solar energy to prevent ice nucleation and melt ice, making them environmentally friendly. Recently, several PADS have been constructed based on various types of photothermal conversion materials, e.g., carbon materials,^[^ ^13^, ^14^, ^15^, ^16^, ^17^ ^]^ 2D materials,^[^ ^18^, ^19^ ^]^ and plasmonic^[^ ^20^, ^21^ ^]^ and magnetic particles,^[^ ^22^, ^23^ ^]^ etc., which can generate heat to effectively melt ice and frost under solar illumination. However, there are still challenges that need to be addressed. For instance, many photothermal nanomaterials cannot be directly produced as hierarchical structured surface for light capture without adding polymer binders. Additionally, while photothermal surfaces exhibit de‐icing capabilities, they often show poor icing delay performance at low temperatures. Especially, most of photothermal anti‐/de‐icing surfaces with hierarchical structures still have limitations, e.g., complex preparation processes, practical application, durability, etc. Therefore, it is highly desirable to develop the new photothermal anti‐/de‐icing surfaces with inherently hierarchical structures that excel in both excellent icing delay and de‐icing performance, which remains a major challenge nowadays.

At present, the researched metal–organic frameworks (MOF) with hierarchical structure and porous crystalline have been extensively researched for applications including photo‐catalysis, supercapacitor, and seawater desalination.^[^ ^24^, ^25^, ^26^ ^]^ However, to our knowledge, the application of MOF‐based materials in photothermal superhydrophobic anti/de‐icing field has not been reported before and its photothermal‐conversion mechanism has also not been investigated. In our work, Copper (Cu)‐MOF material is rationally chosen as a competitive candidate for photothermal material, due to its hierarchical structure and excellent solar‐to‐heat conversion ability.^[^ ^26^, ^27^ ^]^ Thus, we successfully developed a MOF‐based photothermal superhydrophobic anti‐/de‐icing surfaces, i.e., MOF‐based micro‐ and nanostructure surface (MOF‐MNS). An optimal MOF‐M_250_NS exhibits a high solar absorption (> 98%) and achieves a significant temperature increment of 65.5 °C. The superior solar thermal conversion mechanism of the Cu‐MOF has also been validated. The MOF‐M_250_NS shows an ultra‐long icing delay time (≈3960 s). Such outstanding photothermal‐conversion ability, along with low‐temperature hydrophobicity of MOF‐M_250_NS, is able to effectively melt the accumulated ice and frost under 1‐sun irradiation at −20 °C, which is attributed to the dual synergy of the hierarchically structured light trapping and the excellent photothermal capacity of Cu‐MOF. Moreover, the MOF‐M_250_NS exhibits durability and versatility, making it as a favorable candidate for practical applications in a harsh environment.

## 2 Results and Discussion

### 2.1 Design of Superhydrophobic MOF‐M250NS

**Figure 1:**
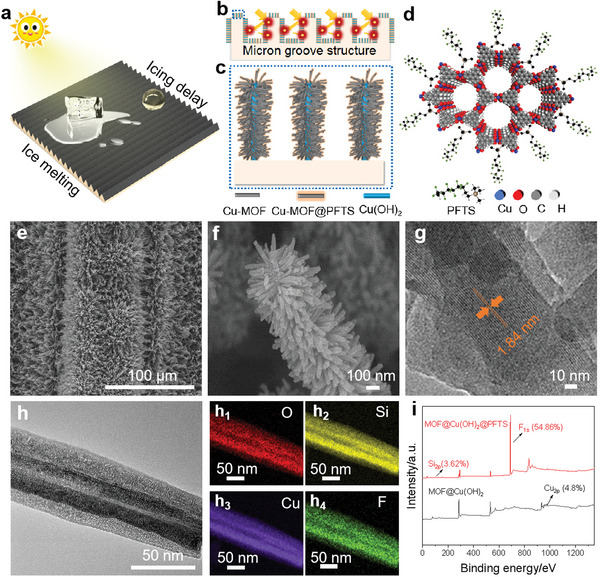
Fabrication and characterization of MOF‐M250NS. a) The prepared MOF‐M250NS possesses outstanding de‐icing ability and ultra‐long icing delay performance. b) MOF‐M250NS surfaces with micron groove structure and hierarchical nanoscale feature and sunlight gets trapped in hierarchical micro/nano groove structure, according to multiple internal reflections within the multilevel Cu‐MOF nanowhiskers. c) Fluorinated Cu‐MOF grown around on Cu(OH)_2_ nanowires, yielding the final tertiary structures on the copper surface with micron groove. d) Schematic diagram of PFTS is successfully grafted onto Cu‐MOF surface at molecular level. e) SEM image and f) High‐magnification SEM image of MOF‐M250NS exhibiting the hierarchical micro/nano structure. The fluorinated Cu‐MOF with length of ≈30–70 nm and diameter of ≈200–300 nm grows around the Cu(OH)2 nanowire. g) HRTEM images for Cu‐MOF@PFTS. The lattice distance of Cu‐MOF is 1.84 nm. h) TEM of Cu‐MOF@PFTS and h1–h4) EDS mapping of the distribution of Cu, Si, F, and O on MOF‐M250NS. Meanwhile, i) XPS survey spectra of Cu‐MOF and Cu‐MOF@PFTS. Additional silicon element with peaks of F1s (54.86%) and Si2p (3.62%) are only found on the Cu‐MOF@PFTS. All these results prove that PFTS has been successfully grafted on the Cu‐MOF.

**Figure** **1**a illustrates the designed MOF‐M_250_NS with photothermal de‐icing capability and ultra‐long icing delay property, resulting from synergistic effect of the hierarchical micro/nano structures of micron grooves and Cu‐MOF with excellent photothermal performance. The MOF‐M_250_NS with micron groove structure has hierarchical nanoscale feature (Figure 1b). It can effectively trap sunlight, which can be reflected multiple times within the internal fluorinated Cu‐MOF nanowhiskers (Figure 1c) until being absorbed completely, leading to superior solar‐heat‐conversion capability. Meanwhile, 1H,1H,2H,2H‐perfluorodecyltrimethoxysilane (PFTS) is also successfully grafted onto Cu‐MOF surface at molecular level (Figure 1d). Lastly, this designed structure can also contribute to the ultra‐long icing delay property on the MOF‐M_250_NS surface. As a result, a representative MOF‐M_250_NS is prepared with a size of up to 10.27 inches (Figure S1, Supporting Information). Scanning electron microscopy (SEM) images also depict the Cu sheet with micron groove structure (Figure S2f, Supporting Information; Figure 4a). Cu(OH)_2_ nanowires are first formed onto Cu sheet with micron groove via the chemical oxidation reaction (Figure S4b,c, Supporting Information). After the growth of Cu‐MOF and fluorination, SEM images clearly exhibit the unique hierarchical micro‐ and nanostructure of the yielded MOF‐M_250_NS (Figure 1e,f; Figure S4d, Supporting Information). The fluorinated Cu‐MOF, with a length of ≈30–70 nm and diameter of ≈200–300 nm, radially grows around the Cu(OH)_2_ nanowire (Figure 1f). The high‐resolution TEM (HRTEM) image also reveals that the lattice distance of Cu‐MOF is 1.84 nm, which is equivalent to (100) plane of Cu‐MOF (Figure 1g). X‐ray diffraction (XRD) pattern of Cu‐MOF further confirms Cu‐MOF successfully grows on Cu(OH)_2_ nanowires (Figure S5, Supporting Information). To gain insight into the fluorinated Cu‐MOF nanostructures, surface morphology and chemical state of the Cu‐MOF@PFTS were analyzed by transmission electron microscopy (TEM) (Figure 1h), energy‐dispersive X‐ray spectroscopy (EDS) (Figure 1h_1_–h_4_) and X‐ray photoelectron spectroscopy (XPS) (Figure 1i). The Cu, O, Si, and F elements are evenly distributed on Cu‐MOF@PFTS surface, indicating that PFTS has been successfully bonded on the surface of Cu‐MOF (Figure S6, Supporting Information). Finally, the as‐obtained MOF‐M_250_NS with a contact angle of 159° and a rolling angle of 4.2° for water droplet of 3 µL exhibits excellent superhydrophobic properties at the ambient temperature (27 °C) (Figure S7b, Supporting Information). Notably, it still maintains a large contact angle value of 145.6° at −5 °C (Figure S7a, Supporting Information), which is much higher than others of MOF‐MxNS. This is mainly attributed to higher roughness (Figure S8a–h, Supporting Information), hierarchical micro‐ and nanostructure, and low surface energy. The MOF‐M_250_NS with low temperature water‐repellent makes it practical for use in cold climates, which can be considered as a robust candidate.

### 2.2 Superior Photothermal Performance of MOF‐MXNS

**Figure 2:**
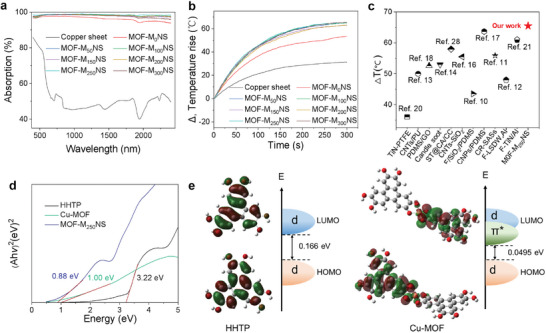
Photothermal performance of MOF‐M_250_NS. a) Light absorption spectra of MOF‐M_X_NS and Cu sheet in the range of solar spectrum (400–2400 nm), which is obtained from 1‐R, where R represents light reflectance. It is noticeable that the absorption of MOF‐M_250_NS is higher than 98% than other MOF‐M_X_NS, MOF‐NS, and copper sheet. b) Temperature rises (Δ*T*, relative to the environmental temperature) of the MOF‐M_X_NS and Cu sheet under the illumination of solar power intensities (1 kW m^−2^). As a result, the temperature rises on all MOF‐M_X_NS exceed 62 °C and are higher than MOF‐M_0_NS (53.4 °C) and copper sheet (31 °C), notably where Δ*T* of MOF‐M_250_NS and MOF‐M_300_NS reaches 65.5 °C. c) Comparison in terms of 1‐sun temperature rise (Δ*T*) between the MOF‐M_250_NS and other reported structured solar thermal de‐icing materials reported recently (Ref. [10, 11, 12, 13, 14, 16, 17, 18, 20, 21, 28]). d) UV−vis‐NIR diffuse reflection spectrum treated with Kubelka−Munk function of MOF‐M_250_NS, HHTP, and Cu‐MOF. Meanwhile, the MOF‐M_250_NS with a low band gap energy (0.88 eV) renders effective NIR absorption and photothermal conversion, compared to the band gap energy of HHTP (3.22 eV) and Cu‐MOF (1 eV). e) The highest occupied molecular orbital (HOMO) and lowest unoccupied molecular orbital (LUMO) for HHTP and Cu‐MOF. Consequently, the energy gap between HOMO and LUMO of Cu‐MOF is greatly reduced to 0.0495 eV much smaller than that in HHTP (0.166 eV). The simulation results clearly explain Cu‐MOF with narrow energy gap can increase NIR absorption, and enhance photothermal‐conversion ability.

In exception to its excellent superhydrophobic performance, the hierarchical structure composed of Cu‐MOF also gives MOF‐M_X_NS excellent absorption and photothermal conversion ability, given by the results of the light trapped effect from the hierarchical micro‐nano structures and solar thermal effect of Cu‐MOF.^[^ ^26^ ^]^ Furthermore, the excellent photothermal‐conversion properties are essential for the material's de‐icing ability. It is worth noting that the light transmittance of all samples with copper substrate is 0% and average diffuse reflectance of MOF‐M_50‐300_NS decreases to less than 3%, where MOF‐M_250_NS has a diffuse reflectance of less than 2% (Figure S9a, Supporting Information). Comparatively, Cu sheet and MOF‐M_0_NS without groove structure show a stronger diffuse reflectance. The black MOF‐M_50‐300_NS surfaces with micron groove structure have hierarchical nanoscale feature, which can effectively trap the sunlight. So, light gets trapped in the hierarchical micro/nano groove structure, leading to multiple internal reflections within the multilevel Cu‐MOF nanowhiskers until being absorbed completely.^[^ ^11^, ^14^ ^]^ As a result, M_50‐300_NS has excellent light absorption (> 97%), whereas that of the MOF‐M_250_NS is higher than 98% (**Figure** **2**a). Owing to higher solar absorption capacity, the surface temperature rise (ΔT) of MOF‐M_250_NS and MOF‐M_300_NS can achieve 65.5 °C for 1‐sun illumination (Figure 2b; Figure S9b, Supporting Information) and they both possess a 74% photothermal conversion efficiency (Note S1 and Table S1, Supporting Information), higher than that of copper sheet and MOF‐M_0‐200_NS. Meanwhile, the equilibrium temperatures of the copper sheet and MOF‐M_X_NS under different solar illumination intensities and solar illumination angles are also shown in Figure S9c,d (Supporting Information). What's more, the temperature rise (Δ*T*) for the MOF‐M_250_NS with hierarchical structure is also outstanding among the structured solar thermal de‐icing material reported recently (Figure 2c).^[^ ^10^, ^11^, ^12^, ^13^, ^14^, ^16^, ^17^, ^18^, ^20^, ^21^, ^28^ ^]^ To gain in‐depth insight into the photothermal mechanism of the Cu‐MOF, we measure the UV‐diffuse reflectance spectra of powdered Cu‐MOF, 2,3,6,7,10,11‐hexahydroxytriphenylene(HHTP) monomer, and MOF‐M_250_NS and estimate their band gap energy by Kubelka‐Munk functions (Figure 2d). The MOF‐M_250_NS with a band gap energy of 0.88 eV exhibits a wider optical absorption range and narrower band gap, compared to between powdered Cu‐MOF (1 eV) and HHTP (3.22 eV). The Cu‐MOF nanowhiskers with such low band gap energy renders effective NIR absorption and photothermal conversion ability. Wherein, the presence of a large abundance of π‐π conjugated structures in Cu‐MOF (Figure S10, Supporting Information) can also synergistically enhance its absorption of near‐infrared light, just as similar effect to other reported MOF.^[^ ^29^, ^30^ ^]^ To further enlighten the effect of molecular structure on the photothermal performance, we have calculated the energy bands of Cu‐MOF optimized partial structure and HHTP by using Gaussian 09 series software (Figure 2e). The energy gap between highest occupied molecular orbital (HOMO) and lowest unoccupied molecular orbital (LUMO) of Cu‐MOF is significantly reduced to 0.0495 eV, which is much smaller than that observed in HHTP. Moreover, it exhibits highly ionized domain electronic features, thus further validating our experimental results. Ultimately, the dual synergy of Cu‐MOF with solar‐thermal‐conversion performance and hierarchical structures with light trapping enables MOF‐M_250_NS to exhibit excellent photothermal conversion capability.

### 2.3 Icing Delay Performance of MOF‐M_250_NS

**Figure 3:**
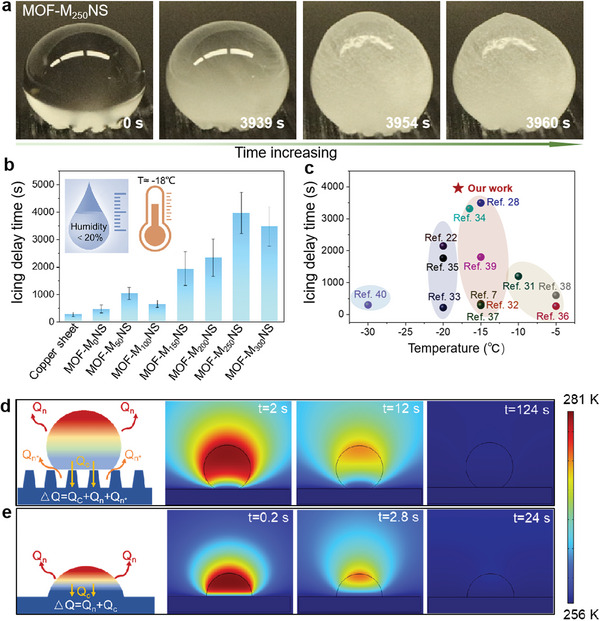
Icing delay performance of MOF‐M_250_NS. a) Optical photograph of water droplet icing process on MOF‐M_250_NS. b) Comparison of icing delay time for 7 µL of water droplets on different MOF‐M_X_NS and copper sheet. The temperature and relative humidity are set to −18 °C and RH<20%. The average icing delay time of the MOF‐M_250_NS with 3960 ± 751 s is the largest among all the tested samples, indicating that it can effectively resist ice formation low ambient temperature conditions. c) Comparison of icing delay time between our MOF‐M_250_NS and other recently reported ice‐delayed surfaces (Ref. [7, 22, 28, 31, 32, 33, 34, 35, 36, 37, 38, 39, 40]). d,e) COMSOL simulations of a water droplet icing process on the surface of MOF‐M_250_NS d) and copper sheet e). From the heat transfer equation: $\Delta Q\;=Q_{c}\;+Q_{n}+Q_{n}^{*}$, where ΔQ represents the heat loss; *Q_C_* is heat conduction; *Q_n_* is heat loss by natural convection;$\;Q_{n}^{*}$ is the heat radiation loss at the three‐phase interface. Obviously, the heat loss of the water droplet on the MOF‐M_250_NS surface is less than that of copper sheet. As shown by the COMSOL simulation results, the time required for the water droplet on the MOF‐M_250_NS surface to cool down to the ambient temperature is 124 s, highly five times that of copper sheet.

As MOF‐M_250_NS has shown outstanding hydrophobic properties at low temperatures, making it widely to be used in the field of passive anti‐icing. Therefore, we record the icing delay time of water droplets on the MOF‐M_250_NS and other copper sheet and MOF‐M_x_NS under the condition of temperature −18 ± 1 °C and RH < 20%. **Figure** **3**a shows the photography of represented droplet in icing process on MOF‐M_250_NS, along with a long icing delay time of 3960 s, which is far better than those of copper sheet and other MOF‐M_X_NS (Figure 3b). More control experiments can be seen in Supporting Information (Figure S11–S14 and Table S2, Supporting Information). Conversely, MOF‐M_250_NS would be more susceptible to ice formation (with a short icing time of 206 s) in high relative humidity environments (e.g., RH ≈90%) (Figure S13 and S14, Supporting Information). Furthermore, MOF‐M_250_NS offers the longest icing delay time (Figure 3c) among the recently reported ice nuclear delayed surface.^[^ ^7^, ^22^, ^28^, ^31^, ^32^, ^33^, ^34^, ^35^, ^36^, ^37^, ^38^, ^39^, ^40^ ^]^ More interestingly, the water droplet on the MOF‐M_250_NS surface maintains a spherical shape before and after icing. It indicates that the hierarchical MOF micro‐nanostructures maintain the droplet in a stable Cassie state throughout the extended icing process. The reason behind this stability lies in rough hierarchically micro‐nano structures that trap a large number of air cushion between the MOF‐M_250_NS surface and the droplet. These air cushions significantly increase the resistance to heat transfer and reduce its contact area, thereby reducing the heat transfer efficiency between water droplets and the interface. Thus, the MOF‐M_250_NS surface can prevent the nucleation of ice and hinder the growth of ice crystals, thus significantly prolonging the icing time.^[^ ^28^, ^33^ ^]^ Moreover, MOF‐M_250_NS with the low thermal conductivity facilitates the icing delay performance, as it blocks heat transfer to delay the formation of icing (Table S3, Supporting Information). To visualize the heat transfer process of droplet on both the MOF‐M_250_NS and copper sheet surface, COMSOL simulations are utilized to observe the heat exchange process of water droplets on the MOF‐M_250_NS surface (Figure 3d; Movie S1, Supporting Information) and copper sheet surface (Figure 3e; Movie S2, Supporting Information) under the simulated icing environment, and corresponding physical models are shown in Figure S15 and Note S2 (Supporting Information). As indicated by the simulation results, the time needed for a water droplet on the surface of MOF‐M_250_NS to cool down to ambient temperature is 124 s, significantly higher than that of the same droplet placed on a plain copper surface. The simulations also confirm that the hindrance of heat transfer between droplet and surface is mainly due to low actual liquid‐solid contact area, owing to the existence of an abundance of air pockets between the two phases. This elevated the free energy barrier for ice nucleation reduces the ice nucleation rate at the contact points.^[^ ^41^ ^]^ To quantify the process of heat loss of droplets on the MOF‐M_250_NS and copper sheet surface and we calculate the heat loss rate of the water droplets on their surface. Thus, the heat loss rate (*η*) of water droplets on the their surfaces throughout the icing process is defined as:

(1) $$\eta \;=\frac{\Delta Q}{t}\;$$

where Δ*Q* represents the heat loss of the water droplet throughout the icing process; t represents the delay in icing of water droplets. According to the setting of the model establishment and related parameters (Figure S16, Notes S3 and S4, and Table S4, Supporting Information), the heat loss rate on the MOF‐M_250_NS surface can be calculated as η_*m*_= 3.0 × 10^−4^ J s^−1^, which is much lower than the heat loss rate on the copper sheet surface ( η_*Y*_= 3.97 × 10^−1^ J s^−1^). This also adequately explains the superhydrophobic surface with hierarchical structure can prolong the icing time of water droplets, making it widely applicable in the anti‐icing field.

### 2.4 Photothermal Defrosting of MOF‐M_250_NS

**Figure 4:**
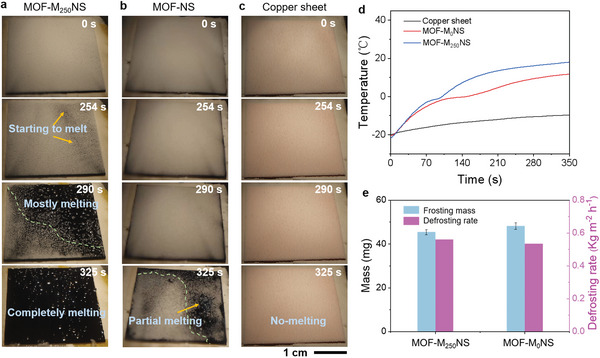
Photothermal defrosting of MOF‐M_250_NS. a–c) Side‐view optical image sequences showing the defrosting process of a) MOF‐M_250_NS, b) MOF‐M_0_NS, and c) copper sheet at a low temperature of −20 °C and RH ≈60% under 1‐sun illumination. The frost on the MOF‐M_250_NS surface starts to melt in 254 s and all frosts on the interface have been completely melted in 325 s. On the contrary, frost on the MOF‐M_0_NS and copper sheet surface is only partly, and almost hardly melted in 325 s, respectively. d) Temperatures of surfaces versus time. The temperature of MOF‐M_250_NS rises to 17.3 °C well higher than the temperature of MOF‐M_0_NS (11.1 °C) and copper sheet (−9.9 °C) during the 325 s of solar thermal de‐frost. e) Mass of frost formation and defrosting rate on the MOF‐M_250_NS to compare that of MOF‐M_0_NS. The frost mass in the MOF‐M_0_NS is ≈48.2 ± 1.5 mg, while that the MOF‐M_250_NS is ≈45.4 ± 1.2 mg under the condition of 5 min of ultrasonic atomization at −20 °C. The simulated ambient temperature of −20 °C under 1‐sun illumination. The average defrosting rate of the MOF‐M_250_NS is 0.561 kg m^−2^ h^−1^ slightly higher than average defrosting rate of MOF‐M_0_NS (0.535 kg m^−2^ h^−1^).

Although our superhydrophobic surfaces can display excellent anti‐icing performance, the entire surface is easily covered by frost in low‐temperature and high‐humidity conditions, which may cause the superhydrophobic surfaces to lose their anti‐icing properties. Additionally, we also investigate the anti‐frost performance of MOF‐M_0_NS and MOF‐M_250_NS. Both surfaces shall eventually be covered with frost, but the MOF‐M_250_NS surface accumulates less frost compared to the MOF‐M_0_NS surface in a given length of time (Figure S17 and Movie S3, Supporting Information). Therefore, defrosting is essential to endow the anti/deicing performance to the materials. We systematically research the defrosting abilities of MOF‐M_250_NS, MOF‐M_0_NS, and copper sheet under sunlight illumination. First, the defrosting process is performed at −20 ± 1 °C and RH ≈60% with 1‐sun illumination. For the MOF‐M_250_NS surface (**Figure** **4**a; Movie S4, Supporting Information), the frost layer starts to melt after being illuminated for 254 s. Afterward, the frost has melted mostly at 290 s, and as‐melted water forms small droplets on the surface. Finally, all frost completely melts, and some water droplets merge and roll off at 325 s, due to the excellent superhydrophobicity of MOF‐M_250_NS. On the contrary, for the control MOF‐M_0_NS and copper sheet, partial melting of frost on the MOF‐M_0_NS surface occurs at 325 s (Figure 4b; Movie S4, Supporting Information) and no frost melting is observed under the sun illumination for copper sheet surface at the same time (Figure 4c; Movie S4, Supporting Information). Meanwhile, we record the temperature fluctuations that each sample undertook during the defrosting process, conducting under 1‐sun illumination (Figure 4d). MOF‐M_250_NS exhibits a significantly faster heating rate of 0.119 °C s^−1^, reaching a surface temperature of 17.3 °C within 325 s. This temperature surpasses that of the copper sheet's equilibrium temperature (−9.9 °C) with a heating rate of 0.031 °C s^−1^, as well as MOF‐M_0_NS (11.1 °C) with a heating rate of 0.096 °C s^−1^. The higher final temperatures and faster heating rates of the MOF‐M_250_NS contribute to its enhanced defrosting efficiency compared to the copper sheet and MOF‐M_0_NS. Even under low temperature and high humidity conditions with 0.5 solar irradiation for 40 min, the surface of MOF‐M_250_NS remains dry (Figure S18, Supporting Information). This demonstrates that the dual synergy of photothermal effect and superhydrophobicity makes the defrosting process more efficient and energy saving.

To quantify the advantages of MOF‐M_250_NS in terms of anti‐frost and defrosting performance, we compare the amount of frost formation and defrost rate of MOF‐M_250_NS and MOF‐M_0_NS (Figure 4e). The frost mass in MOF‐M_250_NS is ≈45.4 ± 1.2 mg, which is less than that of the MOF‐M_0_NS (≈48.2 ± 1.5 mg). The differences in frost mass are the superhydrophobicity of MOF‐M_250_NS enable nucleated microdroplets to dislodge from the tapered microgaps, triggering a spontaneous movement of the droplets, and eventual coalescence‐induced jump caused by the agglomeration of the droplets under supercooling conditions (Figure S17c, Supporting Information). Consequently, the defrosting rate can be calculated, MOF‐M_250_NS exhibits an average defrosting rate of 0.561 kg m^−2^ h^−1^, which is slightly higher than that of MOF‐M_0_NS (average defrost rate of 0.535 kg m^−2^ h^−1^). Thus, MOF‐based photothermal surface exhibits defrost and anti‐frost capabilities.

### 2.5 Photothermal De‐Icing of MOF‐M_250_NS

**Figure 5:**
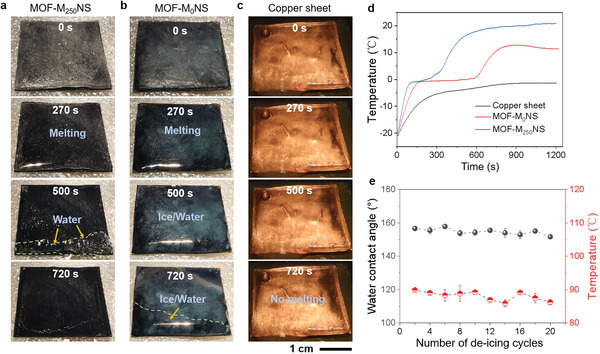
Photothermal de‐icing performances of MOF‐M_250_NS at a low environmental temperature of −20 °C and RH ≈80% under 1‐sun illumination. a–c) Side‐view optical image showing the deicing process of a) MOF‐M_250_NS, b) MOF‐M_0_NS, and c) copper sheet. As for the MOF‐M_250_NS, ice starts to melt at 270 s, then slides down at 500 s, and completely slides off at 720 s. Conversely, the MOF‐M_0_NS surface takes on ice/water mixture in 500 s even 720 s, and the ice layer does not melt on copper sheet surface during the de‐icing process. d) Surface temperature versus time. The temperature of MOF‐M_250_NS rises to 19.2 °C well higher than that of MOF‐M_0_NS (9.3 °C) and copper sheet (−2.2 °C) during the 720 s of solar thermal de‐icing. e) Water contact angle and equilibrium temperature versus de‐icing cycles. Water contact angle and the equilibrium temperature of MOF‐M_250_NS surface under 1 sun are kept at > 150 °C and > 85 °C, respectively, in 20 de‐icing cycles.

We have systematically studied in depth the icing delay and defrosting properties of the MOF‐M_250_NS. Meanwhile, MOF‐M_250_NS exhibits an ice adhesion strength of less than 130 kPa after 10 icing/de‐icing cycle tests (Figure S19, Supporting Information). Despite being prone to icing under relatively high humidity, as the Cu‐MOF with hierarchical structures can yield a light trapping effect, it can effectively convert light energy into heat energy for ice melting. Thus, investigating the photothermal de‐icing ability of MOF‐based materials is highly necessary at −20 °C and RH ≈80%. The ice‐melted ability of MOF‐M_250_NS, MOF‐M_0_NS, and copper sheet are evaluated under 1‐sun illumination. **Figure** **5**a–c shows the melting process of ice on three sample surfaces. For the MOF‐M_250_NS (Figure 5a; Movie S5, Supporting Information), the ice on the MOF‐M_250_NS surface starts to melt at 270 s. Moreover, the ice melts largely, and form water to slide off at 500 s. All of the ice on the MOF‐M_250_NS surface melts within 720 s of illumination and its surface remains almost dry. In comparison, for the control MOF‐M_0_NS and copper sheet, within 720 s illumination, ice‐water mixtures remain on the MOF‐M_0_NS surface and the melting water strongly reflects light, thus greatly reducing solar thermal efficiency (Figure 5b; Movie S5, Supporting Information). On the copper sheet surface, ice does not melt at all time (Figure 5c; Movie S5, Supporting Information). Furthermore, the temperature changes of three samples are monitored during the de‐icing processes (Figure 5d). The MOF‐M_250_NS exhibits a high steady‐state temperature (19.2 °C) with a heating rate of 0.054 °C s^−1^, higher than both the MOF‐M_0_NS (9.3 °C) and copper sheet (−2.2 °C). Moreover, MOF‐M_250_NS achieves a higher de‐icing rate of 5.8 kg m^−2^ h^−1^ than that of MOF‐M_0_NS (4.3 kg m^−2^ h^−1^), compared to previously reported de‐icing materials.^[^ ^18^, ^42^, ^43^ ^]^ Notably, the temperature of MOF‐M_0_NS remains below 0 °C within 522 s, due to the ice‐water mixture on the surface of the sample absorbing much heat, while the copper sheet surface temperature always remains below 0 °C with 720 s. Furthermore, to show the low‐temperature photothermal performance of MOF‐M_250_NS, we conduct practical outdoor photothermal performance tests. The temperature of MOF‐M_250_NS reached a peak of 27.3 °C at an ambient temperature of −4 °C under 0.56 solar irradiation, nearly 4.8×higher than the temperature of the pure copper sheet (5.7 °C) under the same conditions (Figure S20, Supporting Information). The stability of the MOF‐M_250_NS after multiple de‐icing is shown in Figure 5e. The MOF‐M_250_NS surface maintains a water contact angle (>150°) after 20 icing‐deicing cycles and completely removes ice from the surface of MOF‐M_250_NS after each cycle. These are mainly attributed to its superior superhydrophobicity and solar thermal effect. Meanwhile, for MOF‐M_250_NS photothermal performance after after cycle de‐icing test, its temperature maintains above 85.9 °C under 1‐sun illumination. These results demonstrate the stability of MOF‐M_250_NS structure without any damage to its hierarchical micro‐nano structures or changes in chemical structure after 20 icing‐deicing cycles (Figures S21 and S22, Supporting Information). Furthermore, it is well known that sunlight intensity is usually less than 1 sun in winter. To evaluate the de‐icing ability of MOF‐M_250_NS in a simulated real environment, e.g., under 0.7 solar illumination, the ice on the MOF‐M_250_NS surface completely melts 1227 s at a low environmental temperature of −20 °C (Figure S23a and Movie S6, Supporting Information). More surprisingly, when we adjust the sunlight intensity to 0.5 sun, the ice on the MOF‐M_250_NS surface also melt mostly under the same conditions (Figure S23b and Movie S6, Supporting Information). Meanwhile, MOF‐M_250_NS surface reaches temperatures of 9.1 °C and 6.9 °C at 1227 s under 0.7‐ and 0.5‐sun illumination, respectively (Figure S24, Supporting Information). All these results demonstrate that the MOF‐M_250_NS possesses superior and durable ice phobic and de‐icing properties.

### 2.6 The Versatility of the MOF‐M250NS

Icephobic surfaces are crucial in practical applications where durability and versatility are required traits if operating in harsh environment. We test the mechanical robustness, chemical stability, and low temperature self‐cleaning of MOF‐M_250_NS. Following the ASTM D3359‐09 tape tests and sandpaper friction tests, the MOF‐M_250_NS surface has a good adhesion to the substrate with only a few regions being removed, and it retains hydrophobicity even after 130 tape‐peeling cycles (Figure S25a,c_2_, Supporting Information). Moreover, according to the sandpaper friction test, only few regions of Cu‐MOF with hierarchical structure are removed from the MOF‐M_250_NS surface, which also retains hydrophobicity after 20 friction cycles (Figure S25b,c_4_, Supporting Information). These are primarily attributed to the micron‐groove structure that acted as an “armor”, preventing from the removal of nanostructures in tape‐peeling and abrasion‐resistant tests, thus enhancing the material's mechanical robustness (Figure S25d, Supporting Information). Meanwhile, the material remains superhydrophobic and chemical stability after 48 h of corrosion in acid and alkaline solutions. This stability is mainly due to the protective effect of the surface air film on the sample surface (Figure S26, Supporting Information) and the water contact angle of MOF‐M_250_NS maintains > 150° after six months of exposure to the environment, also demonstrating its superior chemical stability (Figure S27, Supporting Information). Finally, MOF‐M_250_NS also exhibits excellent low temperature self‐cleaning performance (Figure S28 and Movie S7, Supporting Information). These results imply that the durable and versatile MOF‐based materials have more opportunities for potential practical applications in the field of anti/de‐icing.

## 3 Conclusions

We design a novel MOF‐based photothermal anti‐/de‐icing material, i.e., MOF‐M_250_NS, by synthesizing and combining micron grooves and Cu‐MOF nanowires structures. In this design, MOF‐M_250_NS achieve the excellent low temperature hydrophobicity to reduce the heat transfer efficiency of the droplet on its surface, and effectively improve the free energy barrier for the formation of ice nuclei. The result leads to robust icing delay time of 3960 s for the 7 µL droplets at −18 °C. As for photothermal performance, MOF‐M_250_NS yields a temperature rise of 65.5 °C under 1‐sun illumination. This enhancement is attributed to the hierarchical micro‐nanostructures of Cu‐MOF, which demonstrates over 98% solar absorption and effectively trapping sunlight, converting it into thermal energy. The accumulated ice and frost on the MOF‐M_250_NS surface can be melted quickly to shed off water droplets, thus maintaining a dry surface. A de‐frosting rate of 0.561 kg m^−2^ h^−1^ and a de‐icing rate of 5.8 kg m^−2^ h^−1^ can be achieved effectively. Additionally, MOF‐M_250_NS exhibits excellent mechanical robustness, chemical stability, and low‐temperature self‐cleaning properties, further enhancing its applicability. This study offers an insight to design of novel materials that can be extended into application realms, e.g., anti‐/de‐icing surfaces of industry, transport, power, etc.

## Conflict of Interest

The authors declare no conflict of interest.

## Supporting information

Supporting Information

Supplemental Movie 1

Supplemental Movie 2

Supplemental Movie 3

Supplemental Movie 4

Supplemental Movie 5

Supplemental Movie 6

Supplemental Movie 7

## Data Availability

The data that support the findings of this study are available in the supplementary material of this article.

## References

[1] Y. Wang , X. Yao , S. Wu , Q. Li , J. Lv , J. Wang , L. Jiang , Adv. Mater. 2017, 29, 1700865. 10.1002/adma.201700865 28452153

[2] J. Liu , C. Zhu , K. Liu , Y. Jiang , Y. Song , J. S. Francisco , X. C. Zeng , J. Wang , Proc. Natl. Acad. Sci. USA 2017, 114, 11285. 29073045 10.1073/pnas.1712829114 PMC5664549

[3] S. Zhang , J. Huang , Y. Cheng , H. Yang , Z. Chen , Y. Lai , Small 2017, 13, 1701867. 10.1002/smll.201701867 29058767

[4] T.‐S. Wong , S. H. Kang , S. K. Tang , E. J. Smythe , B. D. Hatton , A. Grinthal , J. Aizenberg , Nature 2011, 477, 443. 21938066 10.1038/nature10447

[5] W. Niu , G. Y. Chen , H. Xu , X. Liu , J. Sun , Adv. Mater. 2022, 34, 2108232. 10.1002/adma.202108232 34963016

[6] L. Li , S. Khodakarami , X. Yan , K. Fazle Rabbi , A. A. Gunay , A. Stillwell , N. Miljkovic , Adv. Funct. Mater. 2022, 32, 2201521.

[7] L. Wang , Z. Tian , G. Jiang , X. Luo , C. Chen , X. Hu , H. Zhang , M. Zhong , Nat. Commun. 2022, 13, 378. 35046407 10.1038/s41467-022-28036-x PMC8770474

[8] Y. Li , W. Ma , Y. S. Kwon , W. Li , S. Yao , B. Huang , Adv. Funct. Mater. 2022, 32, 2113297.

[9] T. Wang , Y. Zheng , A.‐R. O. Raji , Y. Li , W. K. Sikkema , J. M. Tour , ACS Appl. Mater. Interfaces 2016, 8, 14169. 27192099 10.1021/acsami.6b03060

[10] C.‐H. Xue , H.‐G. Li , X.‐J. Guo , Y.‐R. Ding , B.‐Y. Liu , Q.‐F. An , Y. Zhou , Chem. Eng. J. 2021, 424, 130553.

[11] H. Zhang , G. Zhao , S. Wu , Y. Alsaid , W. Zhao , X. Yan , L. Liu , G. Zou , J. Lv , X. He , Proc. Natl. Acad. Sci. USA 2021, 118, e2100978118. 33903253 10.1073/pnas.2100978118 PMC8106333

[12] N. Li , Y. Zhang , H. Zhi , J. Tang , Y. Shao , L. Yang , T. Sun , H. Liu , G. Xue , Chem. Eng. J. 2022, 429, 132183.

[13] Y. Liu , Y. Wu , Y. Liu , R. Xu , S. Liu , F. Zhou , ACS Appl. Mater. Interfaces 2020, 12, 46981. 32955852 10.1021/acsami.0c13367

[14] S. Wu , Y. Du , Y. Alsaid , D. Wu , M. Hua , Y. Yan , B. Yao , Y. Ma , X. Zhu , X. He , Proc. Natl. Acad. Sci. USA 2020, 117, 11240. 32393646 10.1073/pnas.2001972117 PMC7260993

[15] Z. Xie , H. Wang , Y. Geng , M. Li , Q. Deng , Y. Tian , R. Chen , X. Zhu , Q. Liao , ACS Appl. Mater. Interfaces 2021, 13, 48308. 34587444 10.1021/acsami.1c15028

[16] F. Zhang , D. Xu , D. Zhang , L. Ma , J. Wang , Y. Huang , M. Chen , H. Qian , X. Li , Chem. Eng. J. 2021, 423, 130238.

[17] C. Yang , Z. Li , Y. Huang , K. Wang , Y. Long , Z. Guo , X. Li , H. Wu , Nano Lett. 2021, 21, 3198. 33754736 10.1021/acs.nanolett.1c00452

[18] C. Wu , H. Geng , S. Tan , J. Lv , H. Wang , Z. He , J. Wang , Mater. Horiz. 2020, 7, 2097.

[19] Y. Zhao , C. Yan , T. Hou , H. Dou , H. Shen , ACS Appl. Mater. Interfaces 2022, 14, 26077. 35608175 10.1021/acsami.2c07087

[20] L. Ma , J. Wang , F. Zhao , D. Wu , Y. Huang , D. Zhang , Z. Zhang , W. Fu , X. Li , Y. Fan , Compos. Sci. Technol. 2019, 181, 107696.

[21] W. Ma , Y. Li , C. Y. Chao , C. Y. Tso , B. Huang , W. Li , S. Yao , Cell Rep. Phys. Sci. 2021, 2, 100384.

[22] B. Wu , X. Cui , H. Jiang , N. Wu , C. Peng , Z. Hu , X. Liang , Y. Yan , J. Huang , D. Li , J. Colloid Interface Sci. 2021, 590, 301. 33548613 10.1016/j.jcis.2021.01.054

[23] X. Yin , Y. Zhang , D. Wang , Z. Liu , Y. Liu , X. Pei , B. Yu , F. Zhou , Adv. Funct. Mater. 2015, 25, 4237.

[24] J. Liu , D. Yang , Y. Zhou , G. Zhang , G. Xing , Y. Liu , Y. Ma , O. Terasaki , S. Yang , L. Chen , Angew. Chem., Int. Ed. 2021, 60, 14473. 10.1002/anie.202103398 33826217

[25] Y. Wang , Y. Liu , C. Wang , H. Liu , J. Zhang , J. Lin , J. Fan , T. Ding , J. E. Ryu , Z. Guo , Eng. Sci. 2020, 9, 50.

[26] H. Wang , C. Zhang , X. Ji , J. Yang , Z. Zhang , Y. Ma , Z. Zhang , B. Zhou , J. Shen , A. Du , ACS Appl. Mater. Interfaces 2022, 14, 10257. 35170310 10.1021/acsami.1c20769

[27] W. H. Li , K. Ding , H. R. Tian , M. S. Yao , B. Nath , W. H. Deng , Y. Wang , G. Xu , Adv. Funct. Mater. 2017, 27, 1702067.

[28] Z. Xie , H. Wang , Y. Geng , M. Li , Q. Deng , Y. Tian , R. Chen , X. Zhu , Q. Liao , ACS Appl. Mater. Interfaces 2021, 13, 48308. 34587444 10.1021/acsami.1c15028

[29] Y.‐M. Jo , K. Lim , J. W. Yoon , Y. K. Jo , Y. K. Moon , H. W. Jang , J.‐H. Lee , ACS Cent. Sci. 2021, 7, 1176. 34345668 10.1021/acscentsci.1c00289 PMC8323242

[30] J. Su , N. Xu , R. Murase , Z. M. Yang , D. M. D'Alessandro , J. L. Zuo , J. Zhu , Angew. Chem. 2021, 133, 4839. 10.1002/anie.202013811 33236501

[31] S. Zheng , D. A. Bellido‐Aguilar , X. Wu , X. Zhan , Y. Huang , X. Zeng , Q. Zhang , Z. Chen , ACS Sustainable Chem. Eng. 2018, 7, 641.

[32] P. Wang , T. Yao , Z. Li , W. Wei , Q. Xie , W. Duan , H. Han , Compos. Sci. Technol. 2020, 198, 108307.

[33] W. Li , Y. Zhang , Z. Yu , T. Zhu , J. Kang , K. Liu , Z. Li , S. C. Tan , ACS Nano 2022, 16, 14779. 36103395 10.1021/acsnano.2c05624

[34] J. Y. Ho , K. Fazle Rabbi , S. Khodakarami , X. Yan , L. Li , T. N. Wong , K. Leong , N. Miljkovic , Nano Lett. 2022, 22, 2650. 35245074 10.1021/acs.nanolett.1c04463

[35] Y.‐l. Wu , W. She , D. Shi , T. Jiang , T.‐h. Hao , J. Liu , Q.‐c. Zhang , J. You , R. Y. Li , Compos. Part B: Eng. 2020, 195, 108031.

[36] J. Wei , B. Li , N. Tian , J. Zhang , W. Liang , J. Zhang , Adv. Funct. Mater. 2022, 32, 2206014.

[37] R. Li , S. Tian , Y. Tian , J. Wang , S. Xu , K. Yang , J. Yang , L. Zhang , Small 2022, 19, 2206075. 10.1002/smll.202206075 36534911

[38] C. Zhang , H. Xie , Y. Du , X. Li , W. Zhou , T. Wu , J. Qu , Adv. Funct. Mater. 2023, 33, 2213398.

[39] Z. Zhao , Q. Zhang , X. Song , J. Chen , Y. Ding , H. Wu , S. Guo , ACS Appl. Mater. Interfaces 2023, 15, 3522. 36600550 10.1021/acsami.2c20714

[40] W.‐l. Zhou , T. Wu , Y. Du , X.‐h. Zhang , X.‐c. Chen , J.‐b. Li , H. Xie , J.‐p. Qu , Chem. Eng. J. 2023, 453, 139784.

[41] P. Guo , Y. Zheng , M. Wen , C. Song , Y. Lin , L. Jiang , Adv. Mater. 2012, 24, 2642. 22488894 10.1002/adma.201104412

[42] S. Sheng , Z. Zhu , Z. Wang , T. Hao , Z. He , J. Wang , Sci. China‐Mater. 2022, 65, 1369.

[43] L. Zhang , C. Gao , L. Zhong , L. Zhu , H. Chen , Y. Hou , Y. Zheng , Chem. Eng. J. 2022, 446, 137461.

