# Supplementary material for: Highly Efficient Photothermal Icephobic/de‐Icing MOF‐Based Micro and Nanostructured Surface
Source: Adv Sci (Weinh). 2023 Aug 26;10(34):2304187. doi: 10.1002/advs.202304187 (PMC10700197; doi:10.1002/advs.202304187)
Supplement: Supplementary file 1 — Supporting Information [file ADVS-10-2304187-s008.pdf]

ADVANCED SCIENCE
Open Access

Supporting Information

for *Adv. Sci.*, DOI 10.1002/advs.202304187

Highly Efficient Photothermal Icephobic/de-Icing MOF-Based Micro and Nanostructured Surface

*Lei Zhang, Bingcai Luo, Kun Fu, Chunlei Gao, Xuefeng Han, Maolin Zhou, Tiance Zhang, Lieshuang Zhong, Yongping Hou and Yongmei Zheng**

Supporting Information

Highly efficient photothermal icephobic/de-icing MOF-based micro and nanostructured surface

*Lei Zhang[1], Bingcai Luo[1,2], Kun Fu[3], Chunlei Gao[1], Xuefeng Han[1], Maolin Zhou[1], Tiance Zhang[1], Lieshuang Zhong[1], Yongping Hou[1] and Yongmei Zheng[1]**

[1]Key Laboratory of Bio-Inspired Smart Interfacial Science and Technology of Ministry of Education, School of Chemistry, Beihang University (BUAA), Beijing 100191, PR China

[2]Key Laboratory of Green Chemistry and Technology of Ministry of Education, College of Chemistry, Sichuan University, 29 Wangjiang Road, Chengdu, 610064, People's Republic of China

[3]Key Laboratory of Yangtze River Water Environment, Ministry of Education, Shanghai Institute of Pollution Control and Ecological Security, College of Environmental Science and Engineering, Tongji University, Shanghai, 20092, China

*Corresponding authors: zhengym@buaa.edu.cn

Content

1. Experimental Section
2. Supplementary Figure: Figure S1- S28
3. Supplementary Notes: S1-S4
4. Supplementary Table S1-S4
5. Supplementary Movies: Movie S1- S7

1. Experimental Section

1.1. Materials.

N,N-dimethylformamide (DMF, 99.8%), sodium hydroxide (NaOH, 96%), and ammonium persulfate ($(NH_4)_2S_2O_8$, 98%) were all purchased from Sigma Aldrich. Anhydrous ethanol (C_2H_5OH) and acetone were purchased from Sino Pharm Chemical Reagent Co., Ltd., Shanghai, China. 1H,1H,2H,2H-Perfluorodecyltrimethoxysilane (PFTS) and 2, 3, 6, 7, 10, 11-hexahydroxytriphenylene (HHTP, 95%) were all got from Sigma-Aldrich. Cu sheet (Cu, 1.5mm thick) were purchased from local building materials stores. Hydrochloric acid (HCl, 37%) was purchased from Sino Pharm Chemical Reagent Co., Ltd.. Distilled water was obtained by using a Milli-Q purification system (Millipore Corp., Bedford, MA). All the materials were used without further purification.

1.2. Preparation of MOF-MNS

The MOF-based micro and nanostructured surfaces (MOF-MNS) was first prepared by photolithography. A Cu sheet was cut into the pieces with dimensions of 30 × 30 × 0.15 mm^3, and thoroughly rinsed with acetone, ethanol and distilled water for 15 min in turn. Then, A series of Cu sheet surface with grooved structure could be fabricated. Here, The copper sheet is subjected to a line sweep surface treatment with a laser cycle with a UV laser marking machine, the obtained copper sheet surface contained groove arrays in 30 × 30 mm^2 square size with different widths (50, 100, 150, 200 250 and 300 μm) and micro-ditch with a diameter of 50 μm (Supporting Information, Fig. S2).

Afterward, the Cu sheet with micron groove structure was washed with diluted HCl solution (5 vol %) to remove black CuO powder generated on the sample surface during laser machining process. After washing with ethanol and deionized water and drying. Then, the cleaned Cu sheet was immersed in100 mL mixture solution containing NaOH (2.5 M) and $(NH_4)_2S_2O_8$ (0.13 M) for 20 min at room temperature to grow $Cu(OH)_2$ nanowires on the Cu sheet surface. The resulted $Cu(OH)_2$ nanowires on Cu sheet were thoroughly rinsed with deionized water and ethanol and drying. Subsequently, to make Cu-MOF grow on $Cu(OH)_2$ nanowires, the dried the $Cu(OH)_2$ nanowires on Cu sheet were immersed into a homogenous mixture of DMF (2 mL), distilled water (20 ml) and HHTP (65 mg) in a glass Petri dish and then placed in a pre-heated oven at 70 °C for 20 min. After natural cooling to room temperature, the obtained samples, i.e. MOF-based micro and nanostructured surfaces (MOF-M_XNS), where X represented the micro-ratchet with

periods of ~ 0 μm, ~ 50 μm, ~ 100 μm, ~ 150 μm, ~ 200 μm, ~ 250 μm and ~ 300 μm, was thoroughly rinsed with distilled water and acetone.

After drying, MOF-M_XNS was treated by a chemical vapor deposition process, The -OH groups on the Cu-MOF surface can act as active site to combine with fluoroalkylsilane. Finally, the superhydrophobic photothermal MOF-based materials (MOF-M_XNS) with a micro/nano hierarchical structure was placed in a small beaker with 200 μL PFTS, and treated in a vacuum oven at 100 kPa and 90°C for 1 h to complete the hydrophobic modification.

1.3. Characterizations

1.3.1 Basic test

SEM images were acquired from field emission scanning electron microscopy (JSM-7500F, JEOL, Japan). Transmission electron microscopy (TEM) images were taken on Tecnai F20 (FEI, America). The 3D profile of composite coatings were taken on a 3D optical profiler (Bruker Countor GT K 3D, United States). The phase structure of the nanoparticles was characterized by X-ray diffraction (Lab X XRD-6000, Shimadzu, Japan) with a Cu Kα radiation diffraction (0.15406 nm). XPS spectra was tested from an X-ray photoelectron spectrometer (XPS, Kratos Axis Ultra DLD, England). The optical reflection and transmittance spectra were recorded by a UV-3600 visible spectrophotometer (Shimadzu, Japan). Here, the absorption rate was calculated by subtracting the reflectance from 100%, because the Cu sheets were thick (1.5 mm) enough to avoid transmission. Water contact angles were measured on an OCA40 machine (Data physics Instruments GmbH, Germany). Water droplets of 3 μL were dripped at three different locations on the testing surface. The average and standard deviation of the three data values were recorded. To test the temperature rise under solar illumination, the original Cu sheet and MOF-M_XNS were placed on polystyrene foam. A solar simulator (solar 500) with a xenon light source was used to provide different solar intensity (e.g., 0.5, 0.75 , 1 and 1.5 sun power). The bottom temperature of the sample and ambient temperature are detected by a thermocouple connected to a data acquisition device (JK5008U, Multi-Circuit Temperature Tester). A thermal imager (A655sc, FLIR, America) was used to take IR photos and record surface temperature of samples. The thermal conductivity of the samples was carried out with a thermal conductivity meter (Hot Disk TPS 2500S). The light source in the laboratory is LED cold light source, which did not cause the MOF-M_XNS to produce photothermal effect and affect the experimental results (see Supporting Information, Fig. S3).

1.3.2 The icing delay time Experiments*:*

The icing delay performance was measured by visualizing the icing process and evaluating the icing delay time of a water droplet on the sample placed in a customized freezing chamber composed of a highly transparent quartz vessel. One freezer with ethanol circulation were used for temperature-controlled cooling. Specific operation freezing process was as follows: First, a thermocouple piece was attached to the bottom of the sample, it was placed on a polystyrene foam in a freezing chamber. Then, 10 droplets with a volume of 7 μL were placed on the sample surface at room temperature and N_2 was injected to reduce ambient humidity in the freezing chamber. When the ambient humidity in the freezing chamber is below 20%, cold circulation system with ethanol was opened for cooling chamber temperature. During cooling, when the temperature of the sample substrate is 0°C by observing thermocouple data. The digital cameras and thermocouple was opened for record the icing process (start point). The cold Circulation System was continued until the sample substrate was maintained at −18°C ± 1°C by observing thermocouple data. With time, the droplet on the surfaces were frozen and became non-transparent (end point). When the droplet freezed, it was in an exothermic state and a minor peak of temperature rise appeared on the thermocouple data smoothing curve, this moment represented the icing time. Here, the icing delay time (t_{DT}) was defined as the time taken by the droplet to change from transparent (substrate surface temperature is 0°C) to non-transparent. Meanwhile, the thermocouple records the moment of peak on the substrate surface temperature data was when the droplet freezed, it was used to count the droplet icing time. The icing delay time can be averaged by observing icing behaviors of 10 droplets on surface (see Supporting Information, Fig. S11-14, and also Table S2). Meanwhile, the ice delay performance of droplets on the sample surface also were tested under high humidity condition (RH ~ 90%). The phase transition temperature change of the droplets on the sample surface versus time (see Supporting Infotmation, Fig. S14) was further estimated by using a thermocouple probe adhered to the copper substrate in a small closed cooling chamber.

1.3.3 Ice adhesion test:

The shear strength required to remove ice froms ample is considered to be the ice adhesion strength. Briefly, The sample was placed on and faced to a cuvette (1 × 1 × 3 cm^3) with DI water (1.5 mL). which froze on a cold chamber at -18°C for 1 h with the ambient humidity (RH < 20%). Subsequently, a digital force transducer (Range = 5 N and Resolution = 0.01 N) pushed the ice column to separate it from the

sample surface. The maximum force was recorded to calculate the ice adhesion force. At least three parallel samples were measured to obtain an average value.

***1.3.4 De-icing test**:*

De-icing experiments were carried out in a customized freezing chamber composed of a highly transparent quartz vessel (15 × 15 × 15 cm^3). The freezing circulator containing ethanol was used to cool the freezing chamber of the external circulator (Beijing Xingde Co., Ltd, DFY-5/80 low-temperature thermostat reaction bath (-80°C－96°C)) and the temperature of the ethanol was adjusted by the central control panel to effectively change the temperature inside the external chamber. The temperatures of the quartz vessel and the samples were monitored by thermocouples (JK5008U, Multi-Circuit Temperature Tester) connected to the data acquisition equipment. The sample was placed on polystyrene foam inside the freezing chamber. The ice layer we prepare was made in specific molds (approx. 3 × 3 × 1.5 cm^3) and the quality of the water used on the different samples remained almost the same in a homemade chamber with a temperature of −30°C. Then, sun illumination was started when the sample temperature reached −20°C and RH ~ 80%, and the whole de-icing process was recorded with a digital cameras (Canon PowerShot A650). A solar simulator (solar-500) was used to provide standard 1-sun power (1 kW m^{-2}) during the deicing process. The ice removing rate was calculated by using the following equation: $v_{melt} = \frac{dm_i}{A \times dt}$, where m_i is the mass of melted ice surface, A is the projected surface area, and dt is the time for the ice removing, respectively.

***1.3.5 De-frosting test**:*

Defrosting process was similar to that for the deicing test. A frost layer with approximately 1 mm thickness was produced on the samples surface when placed on the polystyrene foam in the freezing chamber at -30°C and exposed to water steam generated by an ultrasonic nebulizer (DOROSIN, DRS-06A) for 5 min. When the sample temperature was stabilized to −20°C by watching thermocouple data, the light irradiation with intensities of 1 sun was switched on and recorded the entire defrosting process in a high ambient humidity (RH ~ 60%). After each defrosting cycle, the 1-sun temperature rise of the samples was recorded by thermocouple sensing probe.

2. Supplementary Figures

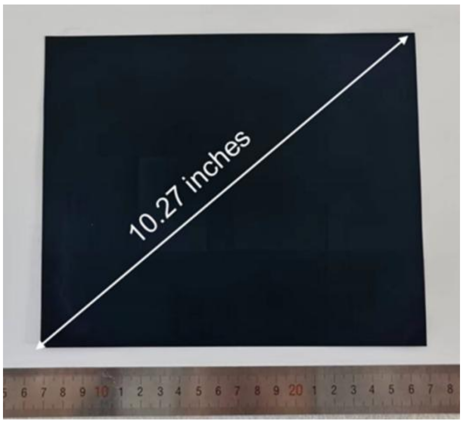

Fig. S1 Top optical image of large area preparation MOF-M_{250}NS. It measures up to 10.27 inches in size.

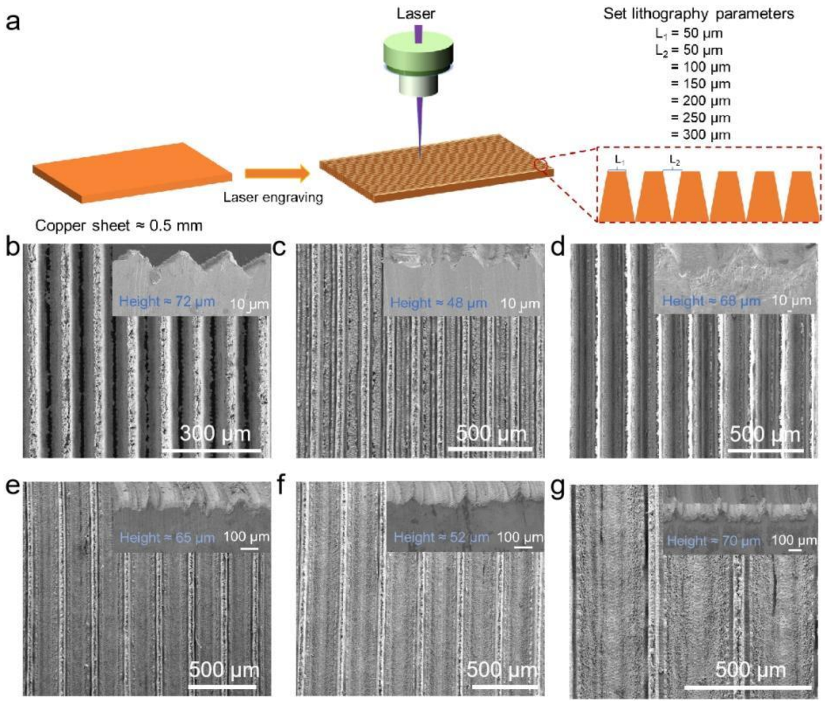

Fig. S2 Preparation of copper sheet surfaces with grooved structures. (**a**) Schematic illustration showing photolithographic copper surface and related parameters setting. (**b-g**) This array is composed by micro-ditch with a diameter of 50 μm and the distance between the edges of two adjacent micron-groove ratchets are ~ 50 μm (b), ~ 100 μm (c), ~ 150 μm (d), ~ 200 μm (e), ~ 250 μm (f), ~ 300 μm (g), respectively. The inset SEM images indicate depth of micron grooves, marked with height ≈ 72 μm, 48 μm, 68 μm, 65 μm, 52 μm, and 70 μm, respectively.

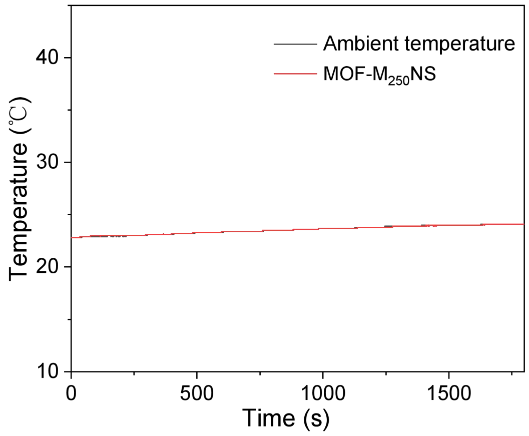

Fig. S3 Temperature variation of the MOF-M_{250}NS under the condition of cold light source (LED lamp) in the laboratory. The surface temperature of the chosen representative MOF-M_{250}NS does not differ significantly from room temperature in a laboratory cold light environment. Thus, the LED light would be insufficient to interfere the photothermal performance of MOF-M_{250}NS.

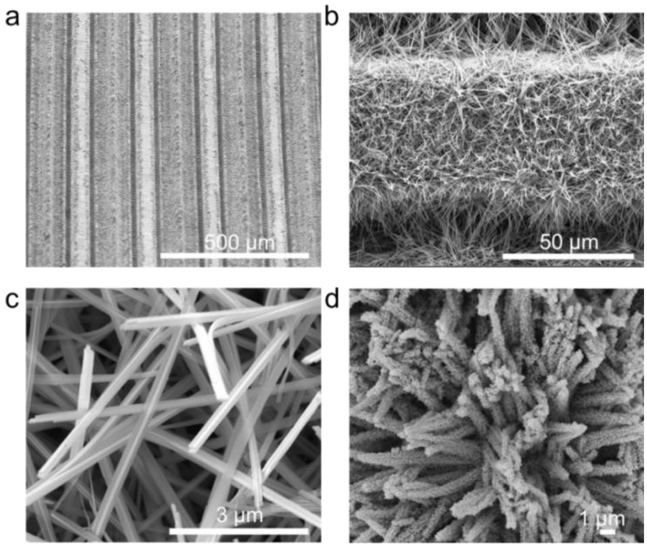

Fig. S4 SEM image during the preparation of MOF-M_{250}NS. (**a**) SEM image of copper sheet with micron grooves. (**b**) SEM image of the $Cu(OH)_2$ nanowires grown on copper sheet with micron grooves. (**c**) High-magnification SEM image of the $Cu(OH)_2$ nanowires with length of around 9 μm and diameter of around 300–500 nm. (**d**) SEM image of monomer HTTP grown on $Cu(OH)_2$ nanowires.

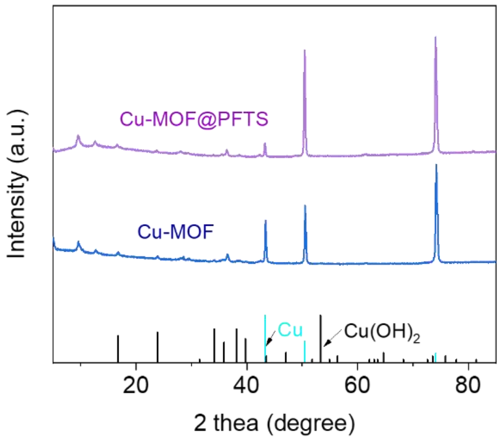

Fig. S5 XRD patterns of Cu-MOF and Cu-MOF@PFTS. The standard peaks for Cu (PDF#04-0836) and $Cu(OH)_2$ (PDF#35-0505). X-ray diffraction (XRD) pattern of Cu-MOF@PFTS further confirms the crystal structure of Cu-MOF nanorods [1-3]. Moreover, the peaks for $Cu(OH)_2$ and Cu can also be observed, indicating that the $Cu(OH)_2$ nanowires backbones and Cu sheet still exist.

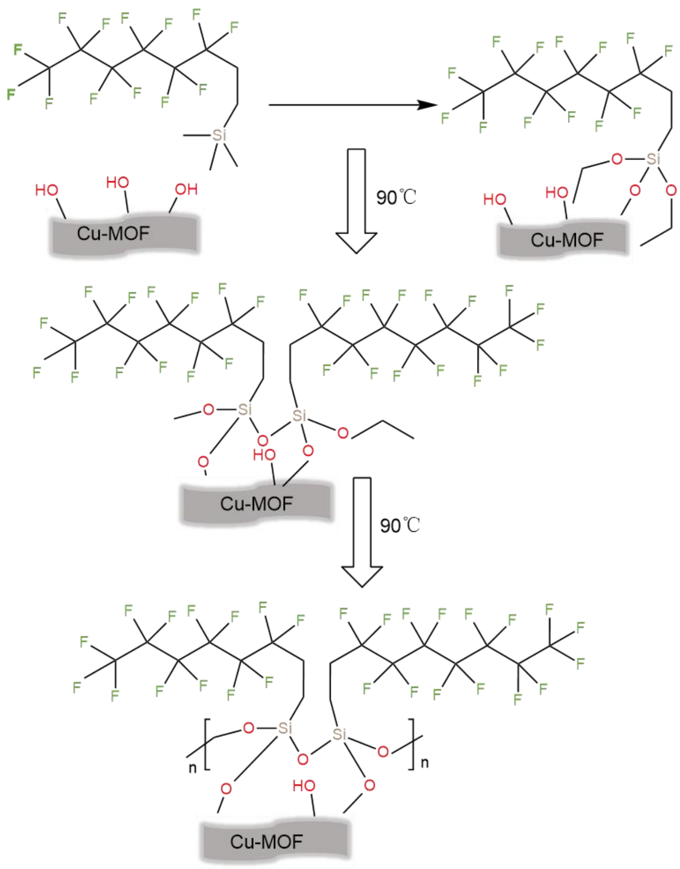

Fig. S6 The Cu-MOF surface modification procedure with PFTS at the molecular level. The results show that PFTS is successfully bounded to the Cu-MOF surface.

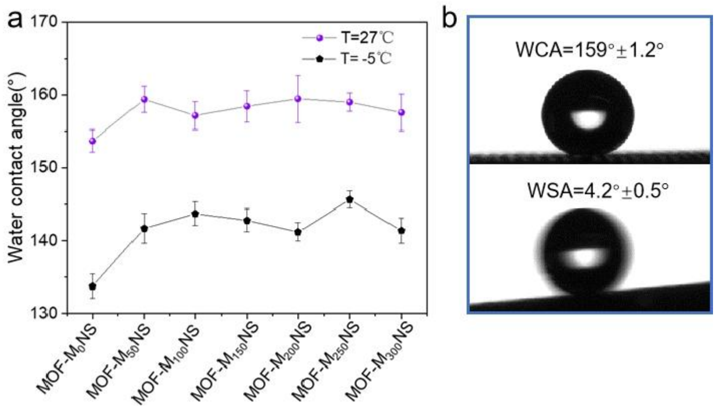

Fig. S7 Water contact angles for different MOF-M_XNS and at 27°C and -5°C and relative humidity (RH ~ 40%). (**a**) Water contact angles on different MOF-M_XNS surfaces. All MOF-M_XNS have contact angles higher than 150° to reach superhydrophobicity at 27°C. MOF-M_{250}NS maintains a large value of 145.6° at -5°C, which is much higher than other MOF-M_XNS. X represents the micro-ratchet with periods of ~ 0 μm, ~ 50 μm, ~ 100 μm, ~ 150 μm, ~ 200 μm, ~ 250 μm and ~ 300 μm, respectively. (**b**) Photo images of droplets on MOF-M_{250}NS. Water contact angle (WCA) of MOF-M_{250}NS is ~ 159° (the top) and the roll angle (WSA) is ~ 4.2° for a water droplet of 3 μL (the bottom), respectively.

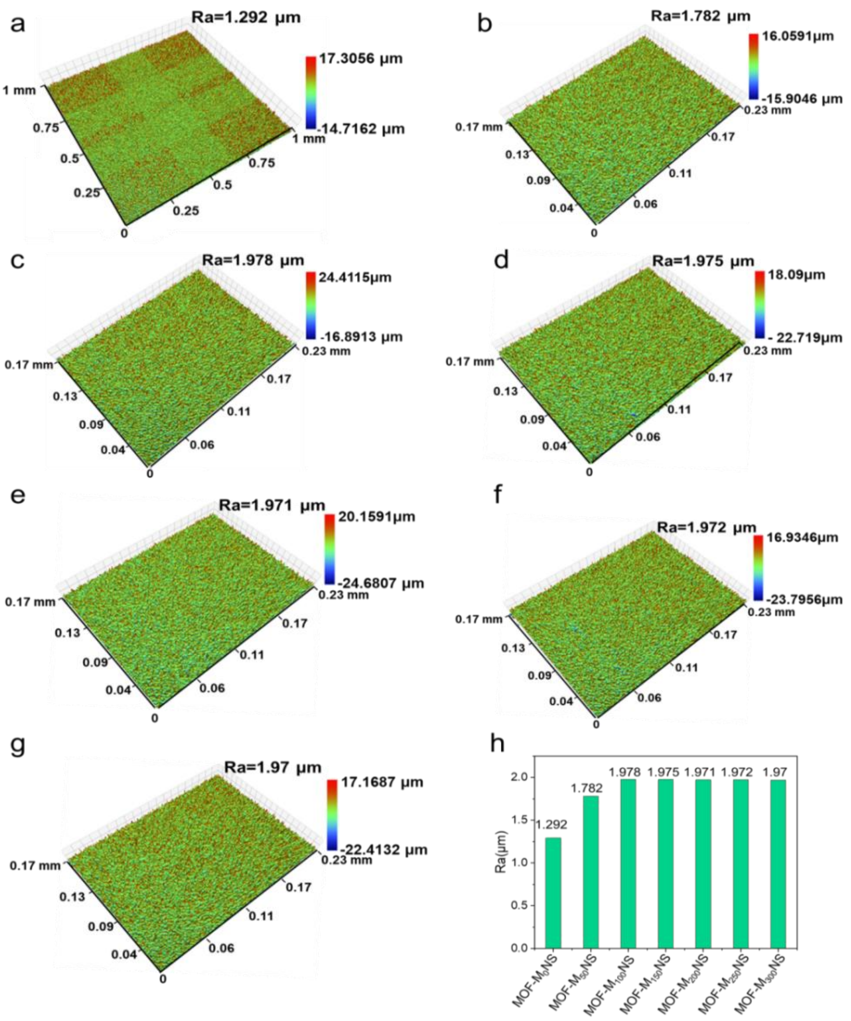

Fig. S8 3D topological image of rough surface of MOF-M$_X$NS. (**a**) MOF-M$_0$NS with Ra=1.292 μm. (**b**) MOF-M$_{50}$NS with Ra=1.782 μm. (**c**) MOF-M$_{100}$NS with Ra=1.978 μm. (**d**) MOF-M$_{150}$NS with Ra=1.975 μm. (**e**) MOF-M$_{200}$NS with Ra=1.971 μm. (**f**) MOF-M$_{250}$NS with Ra=1.972 μm. (**g**) MOF-M$_{300}$NS with Ra=1.97 μm. (**h**) Surface mean roughness (Ra) with MOF-M$_X$NS. When the micron-groove distance is more than 100 μm, the roughness of MOF-M$_{100\text{-}300}$NS remains at about 1.970 μm. X represents the micro-ratchet with periods of ~ 0 μm, ~ 50 μm, ~ 100 μm, ~ 150 μm, ~ 200 μm, ~ 250 μm and ~ 300 μm, respectively.

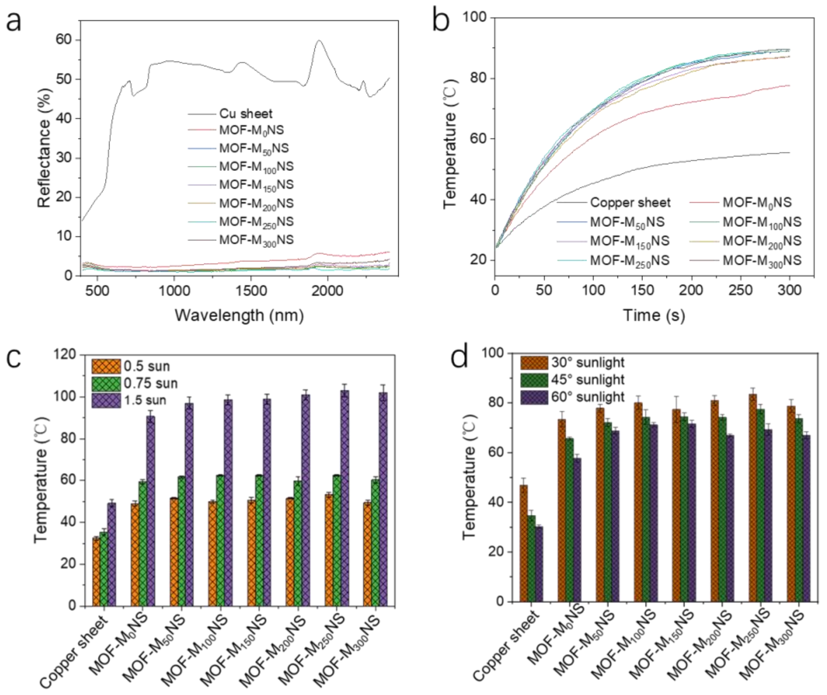

Fig. S9 The diffuse reflectance spectra of copper sheet and MOF-M_XNS. (**a**) Reflectance spectra of MOF-M_XNS and copper sheet. Note that the MOF-M_{250}NS has the lowest reflection (< 2%), compared with other copper sheet and MOF-M_XNS. (**b**) Record of the temperature increasing process of MOF-M_XNS and copper sheet under 1-illumination. The equilibrium temperature of the MOF-M_{250}NS and MOF-M_{300}NS rises sharply, reaching to 89.7℃ after light irradiation for 300 s. (**c**) Surface temperature changes for copper sheet and MOF-M_XNS under different solar illumination of 0.5–1.5 kW m^{-2}. The MOF-M_{250}NS shows a temperature enhancement from 53.2 to 103℃, higher than other copper sheet and MOF-M_XNS. (**d**) Surface temperatures of copper sheet and MOF-M_XNS under 1 sun illumination for 300 s at various incident angles of 30°, 45°, and 60°. The MOF-M_{250}NS always shows a temperature enhancement from 83.5 to 69.3℃, indicating more efficient solar-thermal effect to be compared with other copper sheet and MOF-M_XNS. X represents the micro-ratchet with periods of ~ 0 μm, ~ 50 μm, ~ 100 μm, ~ 150 μm, ~ 200 μm, ~ 250 μm and ~ 300 μm, respectively.

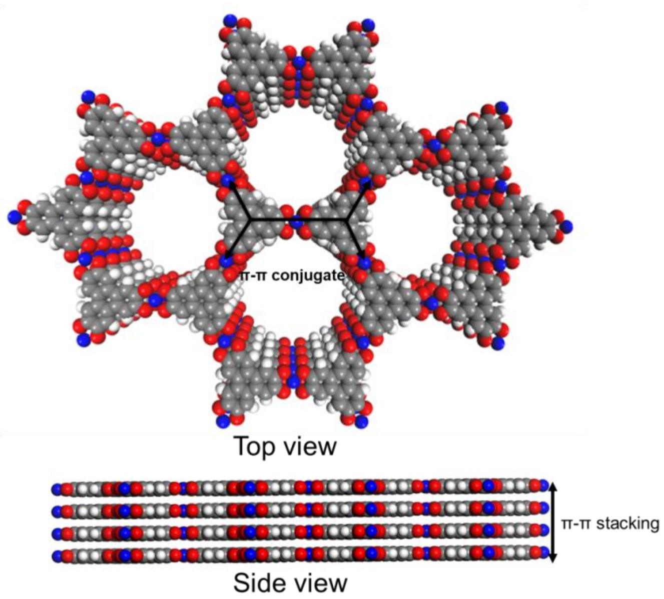

Fig. S10 Schematic diagram of the structure of Cu-MOF. It is obvious that there is π-π stacking structure in Cu-MOF, the π-π conjugated structures in Cu-MOF can also synergistically enhance its absorption of near-infrared light.

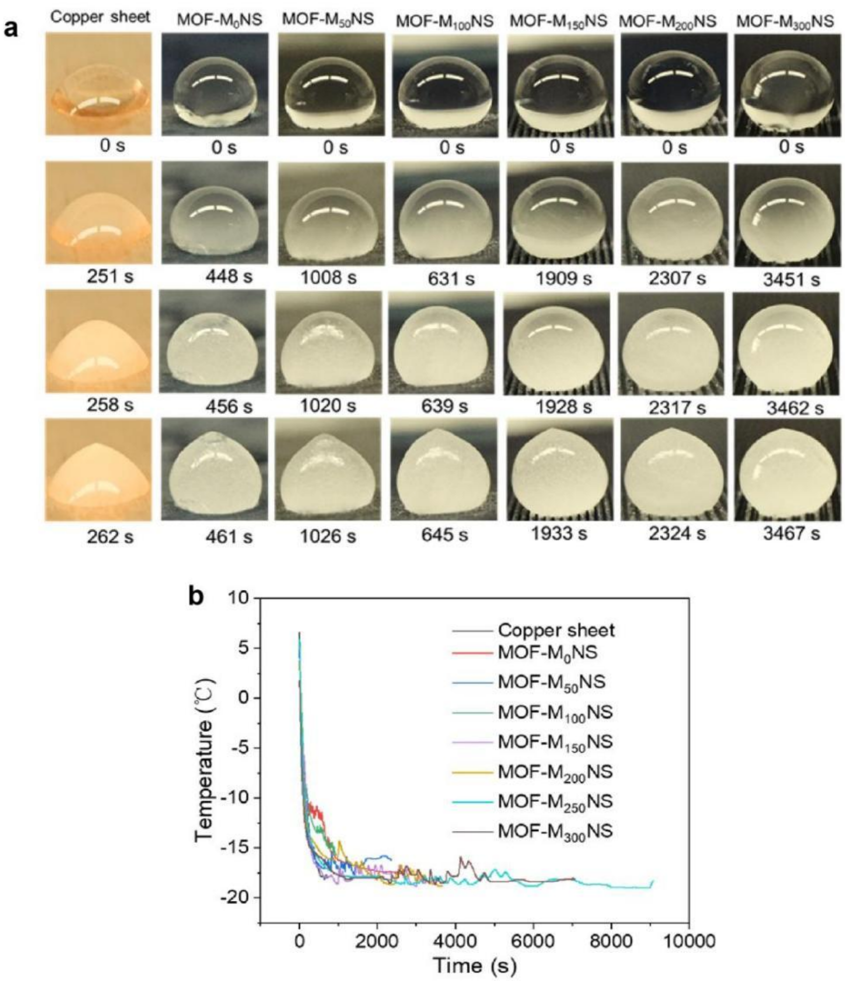

Fig. S11 Test of icing delay time (t_{DT}) on surfaces of samples. (**a**) Optical picture of the icing delay of a droplet (7 μL) on the sample surface. Copper sheet with t_{DT} = 262 s, MOF-M$_0$NS with t_{DT} = 461 s, MOF-M$_{50}$NS with t_{DT} =1026 s, MOF-M with t_{DT} = 645 s, MOF-M$_{150}$NS with t_{DT} = 1933 s, MOF-M$_{200}$NS with t_{DT} = 2324 s and MOF-M$_{300}$NS with t_{DT} = 3467 s. The whole icing delay process is captured by canon cameras, the temperature and humidity are set at -18 ± 1℃ and RH < 20%, respectively. (**b**) Surface temperature change curve of the samples. The small peaks in the temperature profile represent the exothermic process of the droplet during the icing process, thus recording the icing delay time efficiently.

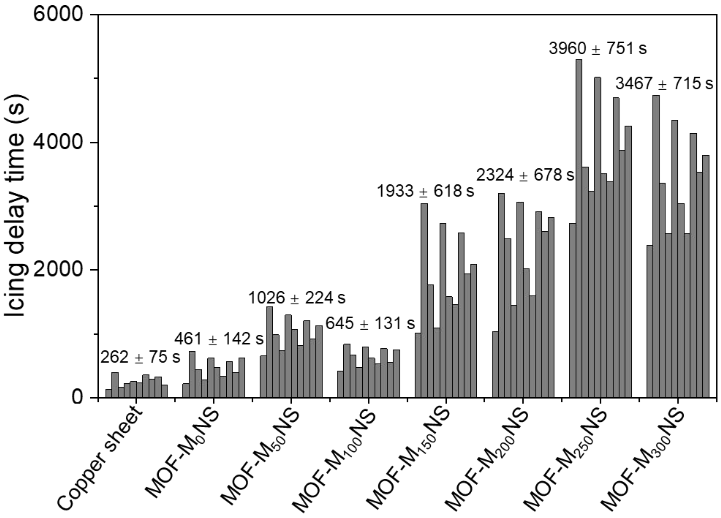

Fig. S12 Distribution of average icing delay times (t_{DT}) for 10 droplets on different surfaces. The test is carried out at -18 ± 1°C and RH < 20%. Volume of droplet is 7 μL. 10 droplets are placed on different locations of the surfaces. The icing delay properties are measured by the average icing time of the 10 droplets on the surfaces, respectively. MOF-M_{250}NS has excellent t_{DT} (3960 ± 751 s), to compare those of copper sheet (t_{DT} = 262 ± 75 s), MOF-M_0NS (t_{DT} = 461 ± 142 s), MOF-M_{50}NS (t_{DT} = 1026 ± 224 s), MOF-M_{100}NS (t_{DT} = 645 ± 131 s), MOF-M_{150}NS (t_{DT} =1933 ± 618 s), MOF-M_{200}NS (t_{DT} = 2324 ± 678 s), and MOF-M_{300}NS (t_{DT} = 3467 ± 715 s).

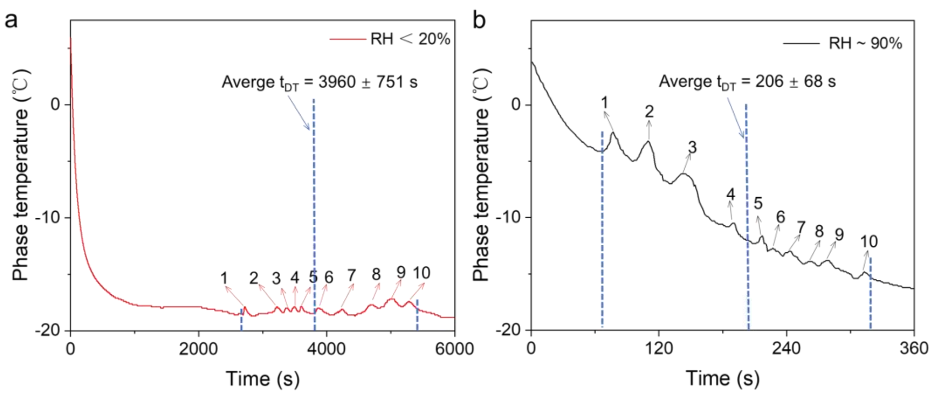

Fig. S13 The phase temperature–time curves of 10 water droplets (7 μL) on MOF-M_{250}NS surface. 10 water droplets are marked with number 1,2,3 … 10. The surface is carried out at -18℃ under both relative humidities, i.e., RH < 20% and RH ~ 90%, rsepectively. The small peaks on curves represent the phase transition process of the water droplet. (**a**) At low relative humidity (RH < 20%). The small peaks appear at time range in Table S2. There is the average icing delay time t_{DT} = 3960 ± 751 s for 10 numbers of droplets. (**b**) At high relative humidity (RH ~ 90%). There is the average t_{DT} = 206 ± 68 s for 10 numbers of droplets. It indicates the high RH would be challenging to obtain excellent icing delay time of a surface.

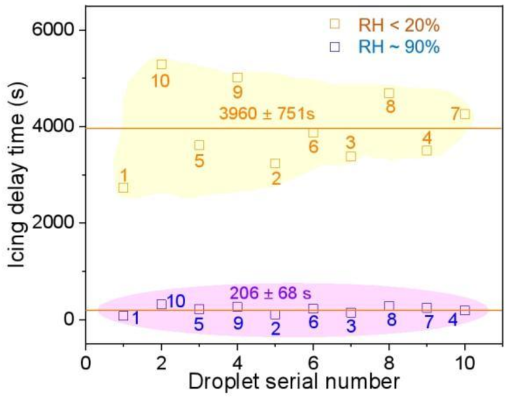

Fig. S14 Distribution of icing delay times for 10 droplets (7 μL) on the MOF-M_{250}NS at low relative humidity (RH < 20%) and high relative humidity (RH ~ 90%) subcooling conditions, respectively. The average icing delay times for 10 droplets (numbered with 1,2,3...10) of the MOF-M_{250}NS is 3960 ± 751 s in RH < 20% and 206 ± 68 s in RH ~ 90%, respectively. The results indicate that superhydrophobic surfaces are prone to icing under high relative humidity. Thus strategy of photothermal deicing is necessary to solve the icing of superhydrophobic surfaces.

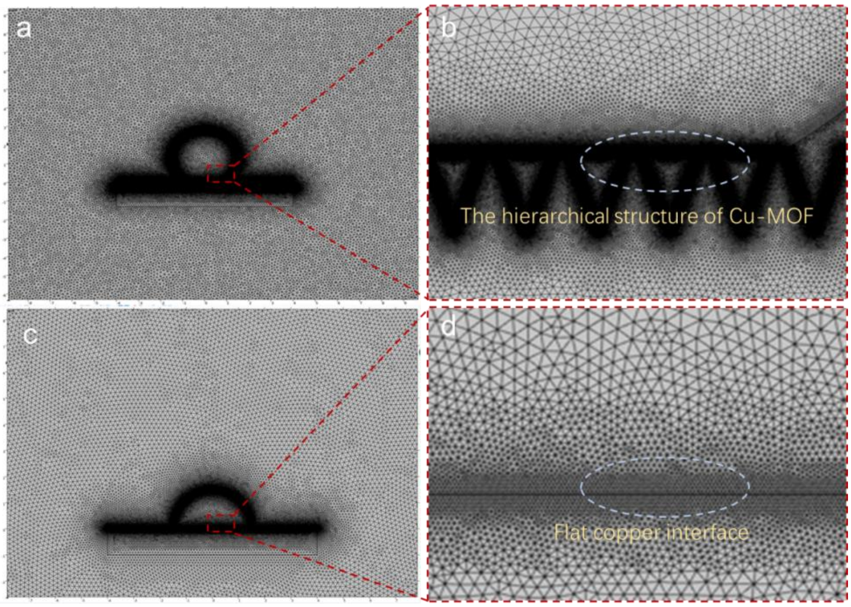

Fig. S15 Modeling the heat transfer of droplets for COMSOL simulation. (**a**) The physical model of a water droplet on MOF-M_{250}NS surface at -18 ± 1℃ and RH < 20%. (**b**) the magnification of the physical model of a water droplet on modeled MOF-M_{250}NS surface under cooling conditions. (**c**) The physical model of a water droplet on copper sheet surface under cooling conditions. (**d**) the magnification of the physical model of a water droplet on modeled copper sheet surface under cooling conditions.

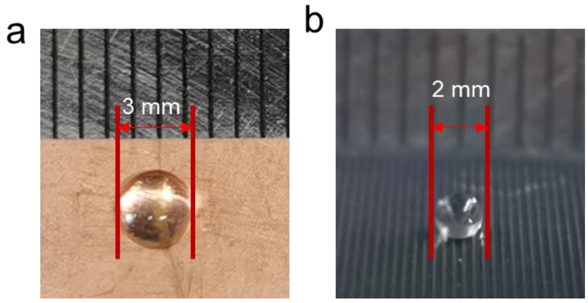

Fig. S16 Differences of droplet diameters on the surface of copper sheet and MOF-M_{250}NS. (**a**) A 7 µL water droplet on the surface of copper sheet takes on a diameter of 3 mm. (**b**) A 7 µL water droplet on the surface of MOF-M_{250}NS takes on a diameter of 2 mm.

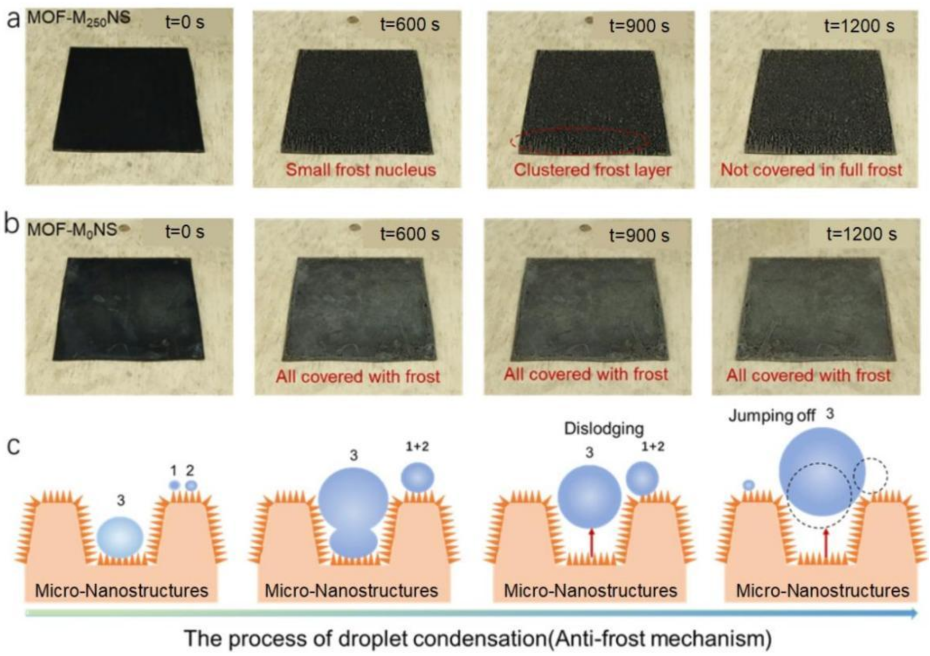

Fig. S17 Anti-frost performance of MOF-M$_0$NS and MOF-M$_{250}$NS. The test is carried out at a relative humidity (RH ~ 60%) and a temperature of - 20°C. (**a**) On surface of MOF-M$_{250}$NS. A small amount of frost nucleus is formed at 600 s and is not completely covered with frost at 1200 s. (**b**) On surface of MOF-M$_0$NS. As a comparison, MOF-M$_0$NS surface has been completely covered with frost at 600 s and a layer of frost is formed at 1200 s. (**c**) Anti-frost mechanism: these grooves with nanostructures created robust superhydrophobic tapered gaps with upward gap-induced laplace pressure. As the small droplets condense and grow, they can enable dislodging of nucleated microdroplets out of the tapered micron grooves and trigger spontaneous merger for finally cohesion-induced jumping.

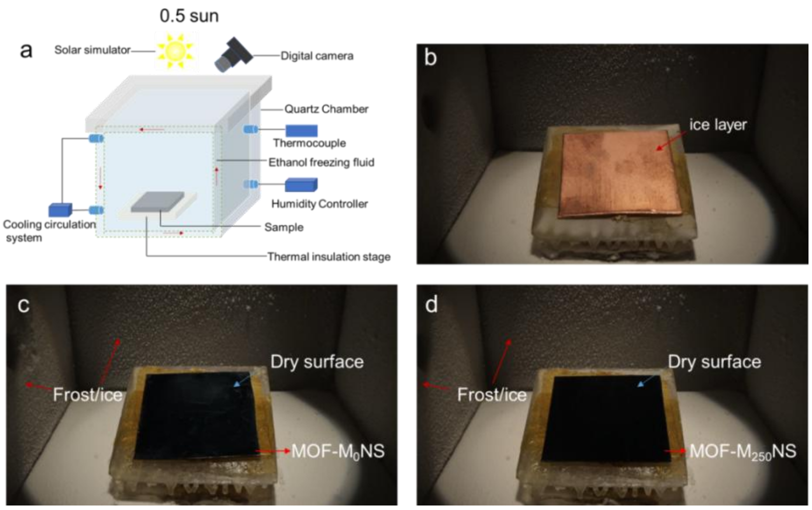

Fig. S18 Photothermal experiment under the low-temperature (-20°C) and high relative humidity (RH ~ 90%) for 40 min. (**a**) Schematic illustration of the experiment setup. (**b**) The surface of the copper sheet is covered with a layer of frost/ice. (**c-d**) The surface of MOF-M_0NS(c) and MOF-M_{250}NS(d) remain completely dry during the entire experime.

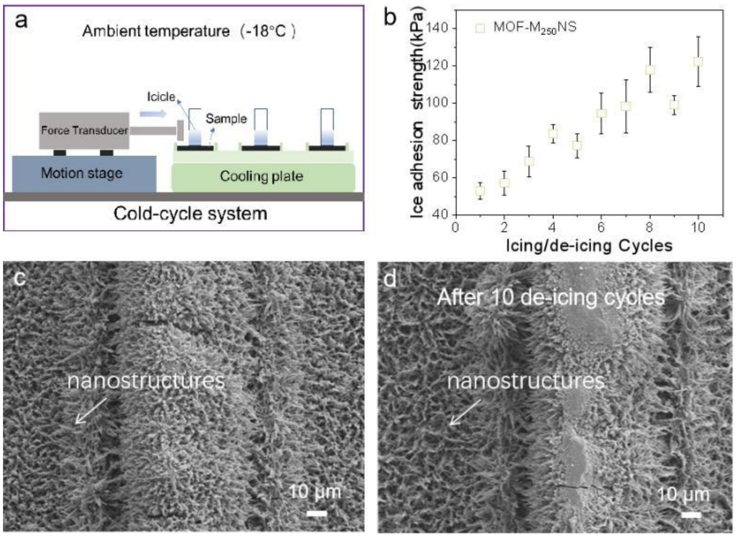

Fig. S19 Ice adhesion test of MOF-M_{250}NS. (**a**) Schematic diagram of ice adhesion strength measurement. (**b**) Variation of ice adhesion strength on MOF-M_{250}NS surface during 10 icing/deicing cycles. The results show that the ice adhesion strength of MOF-M_{250}NS is below 130 kPa after 10 icing/deicing cycles. (**c-d**) Comparison of SEM images of MOF-M_{250}NS before (c) and after 10 de-icing cycles (d). These micron-groove structures act as armor so as to prevent from the removal of nanostructures during the ice adhesion test.

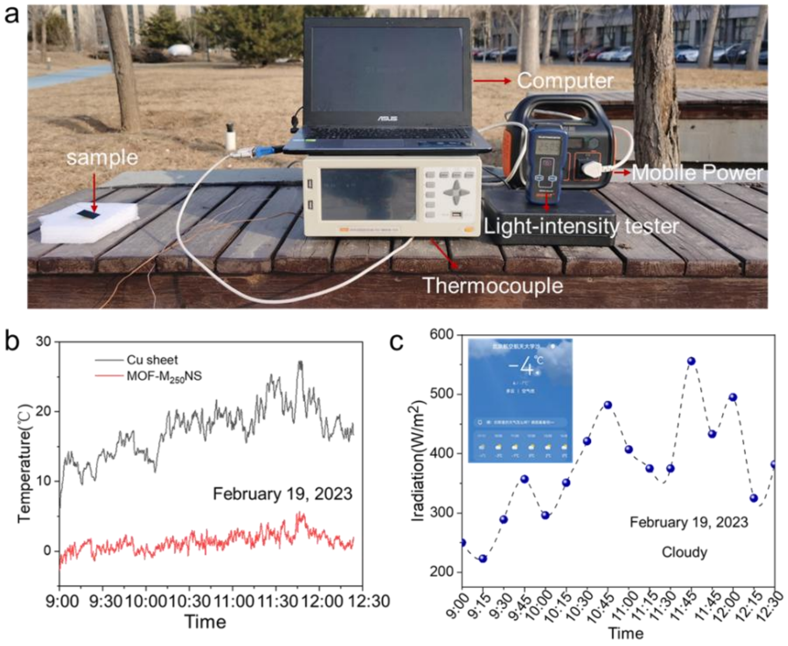

Fig. S20 Outdoor photothermal performance of MOF-M$_{250}$NS. (**a**) Photographs of the experimental setup for outdoor temperature measurements conducted at beihang university on February 19, 2023. (**b**) Variation in temperature of MOF-M$_{250}$NS and copper sheet from 9:00 am to 12:30 pm. The maximum temperature of the MOF-M$_{250}$NS urface reached 27.3°C. (**c**) Solar radiation intensity at 9:00 am−12:30 pm in cloudy weather, it is measured at fifteen-minute intervals. The inset optical image shows the weather and temperature change of beihang university from the weather app on the phone.

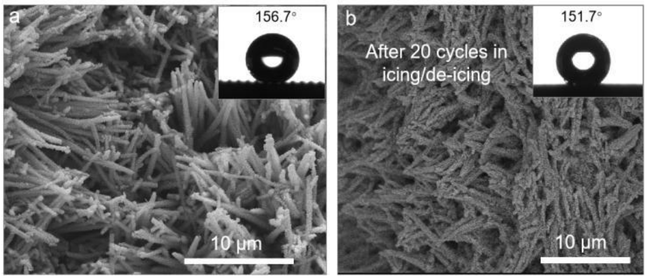

Fig. S21 Stability of morphologies on surface of MOF-M_{250}NS. (**a**) SEM image of surface with water contact angle of 156.7° before icing/deicing. (**b**) SEM image of surface with water contact angle of 151.7° after 20 cycles of icing/deicing (volume of droplet is 3 μL).

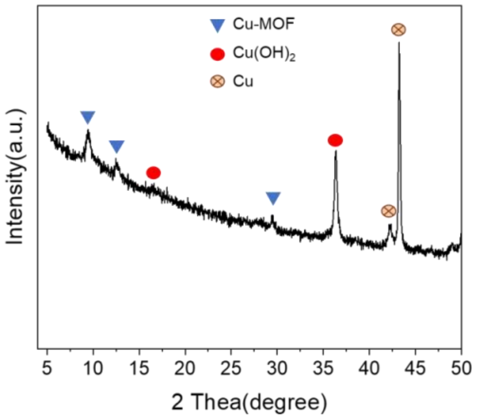

Fig. S22 XRD pattern of MOF-M250NS after 20 cycles of icing/deicing. XRD pattern of the MOF-M250NS after multiple de-icing experiments shows that the characteristic peaks corresponding to the Cu-MOF and $Cu(OH)_2$ nanowires still remain intact, indicating the good stability of MOF-M250NS.

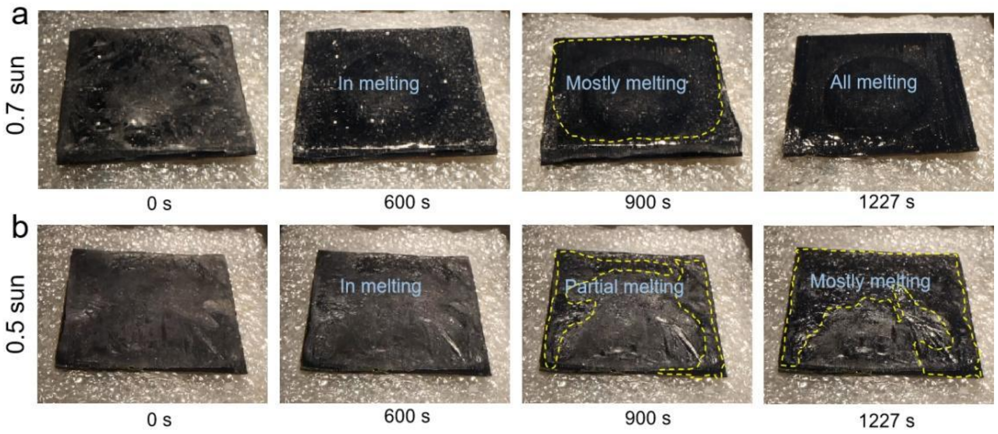

Fig. S23 Photothermal de-icing performances of MOF-M_{250}NS at a low environmental temperature of −20 °C and RH ~ 80% under 0.5-sun and 0.7-sun irradiation. (**a**) For the MOF-M_{250}NS under 0.7-sun illumination. Ice on the sample surface has melted at 900 s and mostly part of the ice has melted at 1227 s. Finally, all ice was completely removed from the sample surface. (**b**) For the MOF-M_{250}NS with 0.5-sun illumination. The partial ice on the sample surface has melted at 900 s and most of the ice has melted at 1227 s. These results indicate that the MOF-M_{250}NS possesses excellent photothermal ability to effectively remove ice at a low temperature environment under less than 1-sun irradiation.

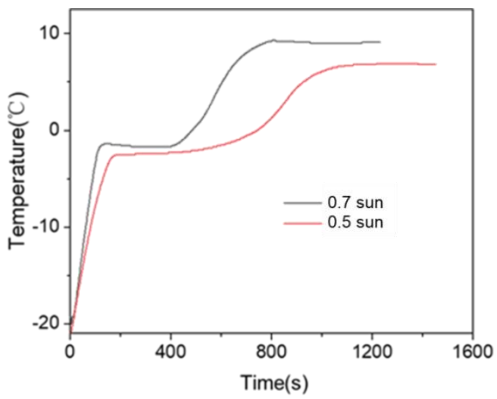

Fig. S24 Curves of temperature change during de-icing of MOF-M_{250}NS under 0.5- solar and 0.7-sun irradiation. The temperature of the sample surface rises to 9.1°C in 1227 s samples under 0.7-sun irradiation is higher than that of 0.5-solar irradiation in the same time.

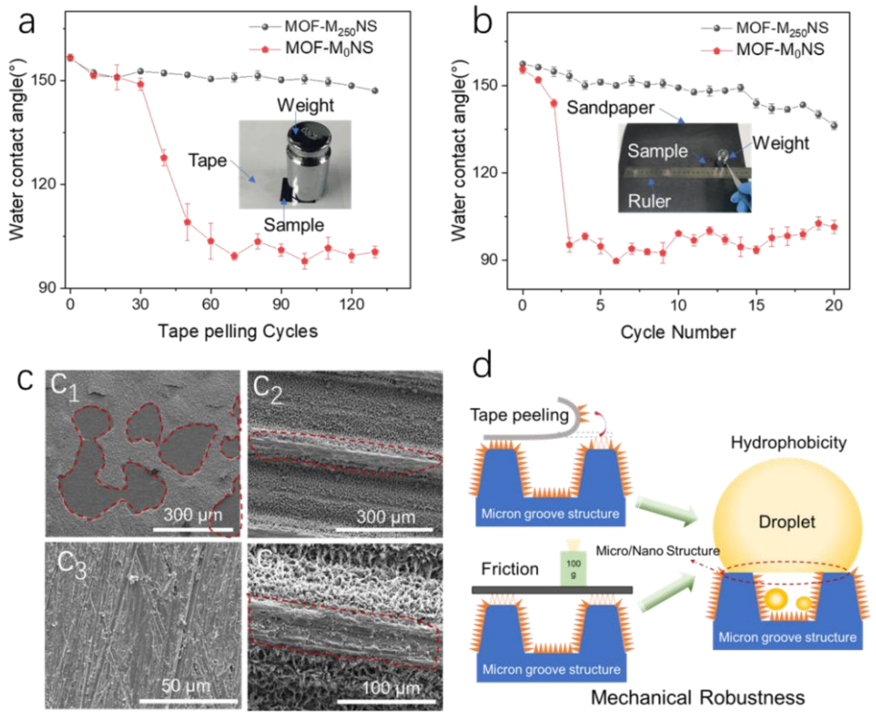

Fig. S25 Mechanical stability of MOF-M$_{250}$NS. (**a**) The inset optical picture of samples with the tape peeling test with 100 g mass on top for uniform 3M tape contact, according to the ASTM D3359-09 standard. The water contact angle of MOF-M$_{250}$NS is kept above 147° much higher than that of MOF-M$_0$NS (100°) after 130 tape-pelling cycles. (**b**) The inset optical picture is schematic diagram of abrasion-resistant test using silicon carbide sandpaper (Grit No. 1300). the abrasion distance in each cycle is 40 cm and the process is repeated 20 cycles. The contact angle of MOF-M$_{250}$NS remains above 136° after 20 friction cycles. For comparison, the contact angle of MOF-M$_0$NS is directly reduced to below 100° after 3 friction cycles. (**c**) SEM photographs of the MOF-M$_0$NS and MOF-M$_{250}$NS after 130 cycles under 100 g mass. Obviously, large-scale peeling of the micro-nanostructure on the surface of MOF-M$_0$NS (c_1) while MOF-M$_{250}$NS surface is only a fallen multi-level structure on the surface of the groove (c_2). The same structural variation also occurs after 20 friction cycles test(c_3-c_4). (**d**) Illustration on mechanical robustness. Although these highly water-repellent nanowires are mechanically fragile, these micron-groove structure act as a “armor” to prevent from the removal of nanostructures in tape-peeling. and abrasion-resistant tests leading to mechanical robustness.

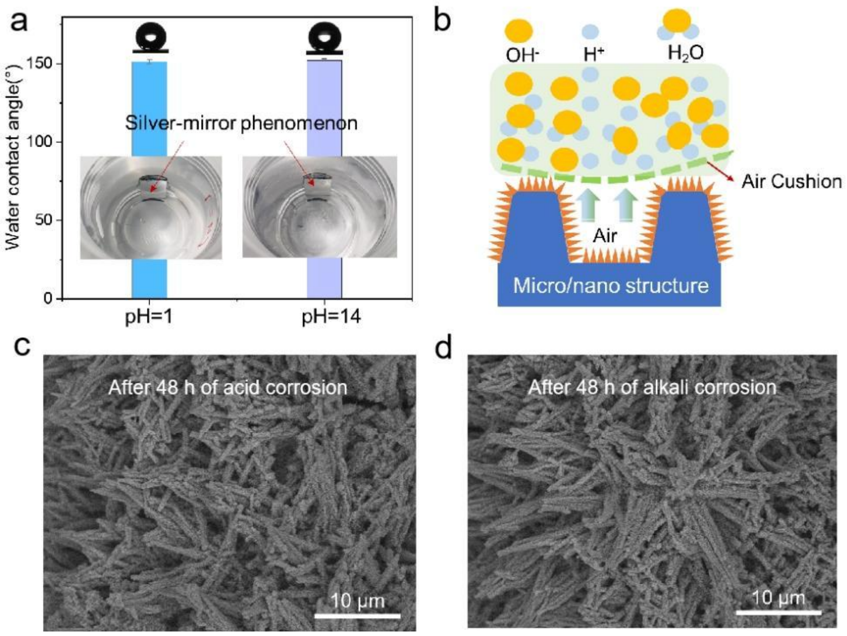

Fig. S26 Chemical robustness of MOF-M_{250}NS. (**a**) Durability of wettability after corrosive solution. Water contact angle of MOF-M_{250}NS as a function of immersion time in H_2SO_4 (pH =1) and NaOH solutions (pH = 14), respectively. The water contact angle of the sample surface is larger than 150° after 48h of solutions corrosion. the inset sample optical images show a silver mirror-like phenomenon. (**b**) Air cushion on surface of MOF-M_{250}NS, which is clearly observed when sample is completely immersed in H_2SO_4 solutions, and NaOH solutions. It acts as additional barriers to effectively prevent the contact of corrosive solutions with the MOF-M_{250}NS and thus improves the chemical resistance of sample. (**c-d**) SEM image indicates the stability of morphologies on surface of MOF-M_{250}NS after 48 h of acid (c) and alkali corrosion (d).

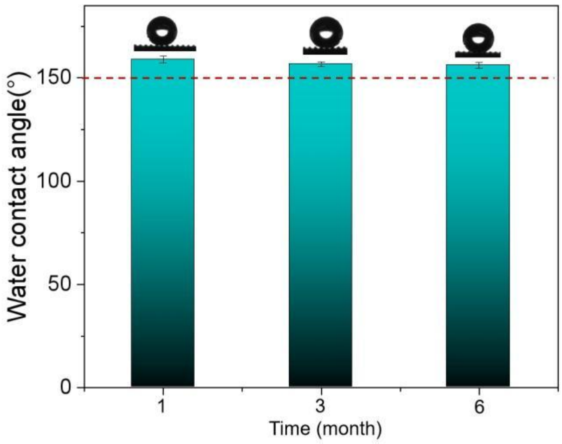

Fig. S27 Superhydrophobic stability of MOF-M_{250}NS. The MOF-M_{250}NS is placed in a transparent glass envelope at temperature of 25 ± 2℃ for 6 months. It retains superhydrophobic properties after six months of exposure to air, demonstrating superior chemical stability.

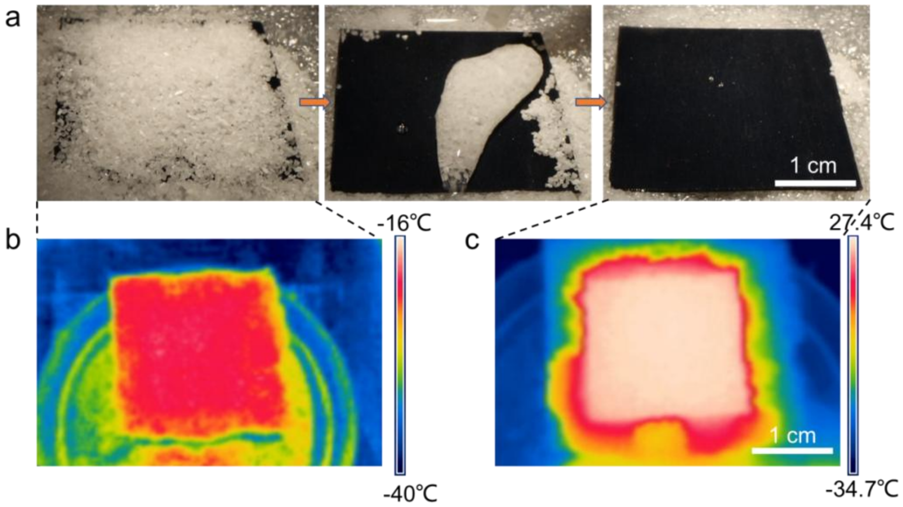

Fig. S28 Self-cleaning ability of MOF-M$_{250}$NS at low temperatures (-40 °C) . (**a**) Optical images of the self-cleaning performance on the sample. The sands are easily taking away by the rolling water droplet. (**b-c**) Infrared camera images of the MOF-M$_{250}$NS under 1 sun before (b) and after (c) self-cleaning. These indicate that the sample has excellent low temperature self-cleaning ability.

3. Supplementary Notes

Note S1. Calculating the photothermal conversion efficiency of copper sheet and M_XNS

We first investigated the photothermal conversion efficiency of all samples, η, which was defined as the ratio of the heat generated by the photothermal conversion of the material to the input of solar power, and the calculation formula is as follows:[4-5]

$$\eta = \frac{Q}{q \times A} \tag{S-1}$$

where Q is the heat generated by the light-to-heat conversion of the material, q represents the light intensity of simulated sunlight (1 kW/m^2), A is the surface area (A = 3.35 cm^2) of the all samples.

When the surface temperature of copper sheet and MOF-M_XNS became stable, the heat generated by the substrate was in balance with the heat dissipation to the surroundings, which could be described by

$$Q = Q_s = h \times A \times (T_{sub} - T_{surr}) \tag{S-2}$$

where Q_s is the heat dissipation to the surrounding environment, h is the convective heat transfer coefficient of the substrate, A is the heat transfer area, T_{sub} is equilibrium temperature of the sample, and T_{surr} is temperature of the sample in the environment. The convective heat transfer coefficient h can be calculated by the following equation:

$$h = \frac{Nu \times \lambda}{L} \tag{S-3}$$

where L is the characteristic length of heat transfer area (L=1.83), λ is thermal conductivity of air, and Nu is the Nusselt number, which can be calculated by the following equation.

$$Nu = 0.54 \times (G_r \times P_r)_m^{\frac{1}{4}} \tag{S-4}$$

Where $G_r = \frac{g a_v \Delta T L^3}{v_2}$ is Grashof number, $P_r = \frac{v}{a}$ is Prandtl number, the subscript m represents qualitative temperature, which can use arithmetic average temperature of the boundary layer ($T_m = \frac{T_{sub}+T_{surr}}{2}$). Where a_v is expansion coefficient of gas, for ideal gases ($a_v = \frac{1}{T}$), T is the average of the sum of the initial ambient and equilibrium temperatures of the sample, g is gravitational acceleration (9.8 m/s^2), ΔT is value of the difference in sample from room temperature to equilibrium temperature, v is the viscosity coefficient, a is the thermal diffusivity. We calculated the photothermal conversion efficiency of copper sheet and MOF-M_XNS, and the relevant data are shown in the Table S1.

Note S2. The physical model of a water droplet on copper sheet and MOF-$M_{250}NS$ surface under cooling conditions.

The COMSOL simulation methods were performed, and the detailed method was explained as follows: two models were established to study heat transfer process of water droplet on the hydrophobic surface (flat surface) or superhydrophobic surface under the cold simulation conditions by the COMSOL Multiphysics software. Before simulations, the following some assumptions were set here:

(1) The volume of water droplet is set 7 under the icing process and the final ambient temperature is set to -18°±1°C. We neglected the evaporation of water and sublimation of ice in the cooling conditions.

(2) The water contact angle of the superhydrophobic surface was 159°, and the water contact angle of the hydrophobic surface was 92°. In fact, the contact angle of a water droplet in a cold environment is a dynamically decreasing trend on a superhydrophobic or hydrophobic surface, but it is assumed that the contact angle does not change during the whole icing process in our model. We also set the thermal conductivity and density of the material (e.g. water, air and copper)

(3) The classical heat and mass transfer equations were still feasible.

$$\frac{\partial(\rho C_p T)}{\partial t} + \nabla(\rho C_p u T) = \nabla[\nabla(kT)] \tag{S-5}$$

Note S3. **Calculating the heat loss rate of water droplets on the MOF-M250NS and copper sheet surface throughout the icing process.**

We define this heat transfer system based on a simple heat balance equation as followings:[6-7]

$$\Delta Q = Q_c + Q_n + Q_n^* \tag{S-6}$$

Where ΔQ represents the heat loss of the water droplet throughout the icing process; Q_C is the heat loss by heat conduction at the two-phase interface; Q_n is heat loss by natural heat convection. Q_n^* is the heat loss in the solid-liquid–air contact area between the three-phase interfaces owe to heat radiation.

For the water droplets on the surface of MOF-M_{250}NS, according to Fourier's law, Newton's law of cooling and Stefan–Boltzmann's law, the equation of the heat transfer process can be defined as:

$$\Delta Q_m = 2\pi\alpha R^2(1 + sin\beta)(T_d - T_s) + 2C_n\pi R^2(1 - sin\beta)\left[\left(\frac{T_d}{100}\right)^4 - \left(\frac{T_{Sub}}{100}\right)^4\right] + \lambda S_{AC}\left(\frac{T_d - T_{Sub}}{x}\right) \tag{S-7}$$

Where α is natural convection heat transfer coefficient; C_n is the radiative heat transfer coefficient; β is the water contact angle of the droplet at ambient temperature; R is the radius of the sphere of the droplet; λ is the thermal conductivity of the MOF-M_{250}NS; T_d represents the initial temperature of the droplet; T_s is the temperature of the sample; T_{Sub} is the surrounding ambient temperature; x is the thickness of the material; t_m is the time taken for the droplet to start icing from its initial state. S_{AC} is contact area of water droplets with the surface.

So, the heat loss rate of droplets on the MOF-M_{250}NS surface throughout the icing process is defined as:

$$\eta_m = \frac{\triangle Q_m}{t_m} \tag{S-8}$$

For the water droplets on the copper sheet surface, there exists not hierarchical structure, the liquid-solid contact area is significantly larger than that of MOF-M_{250}NS. Thus, eqs (S-7) and (S-8) are redefined as:

$$\Delta Q_Y = 2\pi\alpha R^2(1 - sin\beta)(T_d - T_s) + \lambda S_{AY}\left(\frac{T_d - T_{Sub}}{x}\right) \tag{S-9}$$

$$\eta_Y = \frac{\triangle Q_Y}{t_Y} \tag{S-10}$$

Where η_Y is the heat loss rate of the water droplet on the copper sheet surface; t_Y is the icing time of the water droplets on the sample surface; S_{AY} is contact area of water droplets with the surface.

Note S4. **Calculation of the contact area between the droplet and the surface of MOF-M_{250}NS.**

We calculate the ideal actual contact area of MOF-M_{250}NS (S_{AC}) with the water droplets (7 μL) based on Fig. SI. For the S_{AC}, it can be seen from Fig. SI-I and SI-II:

$$r = R\times\cos(159\text{-}90)° = 1000\times\cos(159\text{-}90)° \approx 358\ \mu m \tag{S-11}$$

$$h = \sqrt{r^2 - 275^2} \tag{S-12}$$

$$\text{Actual contact area } S_{AC} \approx 4\times(25\times358+50\times229) = 81600\ \mu m^2 \tag{S-13}$$

For the S_{AY}, it can be seen from Figure S12a:

$$\text{Actual contact area } S_{AY} \approx \pi r^2 = 1500^2 \times \pi = 7068583\ \mu m^2 \tag{S-14}$$

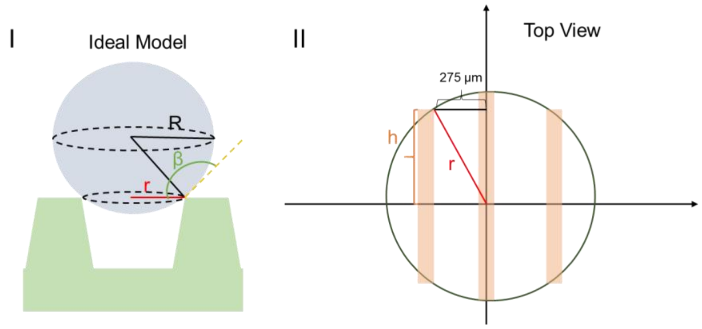

Fig. SI Ideal modeling of liquid droplets on MOF-M_{250}NS surface. (**I**) The ideal heat transfer model of water droplets on superhydrophobic surface for MOF-M_{250}NS, where R represents diameter of droplet, r represents diameter of droplet in contact with superhydrophobic surface and β represents contact angle. (**II**) Top view of droplets on superhydrophobic surface of MOF-M_{250}NS.

Supporting References:

1. J. Liu, D. Yang, Y. Zhou, G. Zhang, G. Xing, Y. Liu, Y. Ma, O. Terasaki, S. Yang, L. Chen, *Angew. Chem. Int. Ed.* **2021**, *60*, 14473.
2. W. H. Li, K. Ding, H. R. Tian, M. S. Yao, B. Nath, W. H. Deng, Y. Wang, G. Xu, *Adv. Funct. Mater.* **2017**, *27*, 1702067.
3. M. Wang, Z. Zhang, Y. Wang, X. Zhao, X. Men, M. Yang, *ACS Appl. Mater. Interfaces* **2020**, *12*, 25512.
4. Z. Xie, H. Wang, M. Li, Y. Tian, Q. Deng, R. Chen, X. Zhu, Q. Liao, *Chem. Eng. J.* **2022**, *435*, 135025.
5. H. Li, R. Chen, X. Zhu, Q. Liao, D. Ye, Y. Yang, W. Li, D. Li, Y. Yang, *Ind. Eng. Chem. Res.* **2021**, *60*, 3758.
6. M. Schremb, S. Borchert, E. Berberovic, S. Jakirlic, I. V. Roisman, C. Tropea, *Int. J. Heat Mass Tran.* **2017**, *109*, 971.
7. Y. Sun, Y. Wang, W. Liang, L. He, F. Wang, D. Zhu, H. Zhao, *ACS Appl. Mater. Interfaces* **2022**, *14*, 49352.

4. Supplementary Tables:

Table S1 Photothermal conversion efficiency of copper sheet and MOF-M_XNS

	Copper sheet	MOF-M_0NS	MOF-M_{50}NS	MOF-M_{100}NS	MOF-M_{150}NS	MOF-M_{200}NS	MOF-M_{250}NS	MOF-M_{300}NS
T_{surr} (℃)	24.2	24.2	24.2	24.2	24.2	24.2	24.2	24.2
ΔT(℃)	31.4	53.4	64.9	64.9	62.9	63.1	65.5	65.5
T_m (℃)	39.9	50.9	56.65	56.65	55.65	55.75	56.95	56.9
T_{sub} (℃)	55.6	77.6	89.1	89.1	87.1	87.3	89.7	89.6
T (K)	312.9	323.9	329.65	329.65	328.65	328.75	329.95	329.9
λ(W/m k)	276	283	288	288	287	287	288	288
$v\times10^6$(m^2/s)	16.94	18.05	18.62	18.62	18.52	18.53	18.65	18.65
$a\times10^6$(m^2/s)	24.26	25.85	26.68	26.68	26.54	26.55	26.73	26.72
$a_v\times10^3$(K^{-1})	3.20	3.09	3.03	3.03	3.04	3.04	3.03	3.03
P_r	0.698	0.698	0.698	0.698	0.698	0.698	0.698	0.698
$G_r\times10^{-4}$	2.74	3.97	4.45	4.45	4.37	4.38	4.47	4.46
Nu	6.35	6.97	7.17	7.17	7.14	7.14	7.18	7.17
h(W/m^2·K)	9.58	10.78	11.28	11.28	11.20	11.21	11.30	11.28
Q(mW)	100.8	192.8	245.2	245.2	236.0	236.9	248.0	247.1
η(%)	30.1	57.5	73.1	73.1	70.4	70.7	74	74

Table S2 Time distribution intervals of phase transition temperatures for 10 droplets

Water droplet （number）	RH < 20% （Time statistics）	RH ~ 90% （Time statistics）
1	2734 s	77 s
2	3237 s	110 s
3	3380 s	144 s
4	3509 s	189 s
5	3613 s	217 s
6	3876 s	228 s
7	4256 s	243 s
8	4694 s	262 s
9	5017 s	280 s
10	5291 s	313 s
**Average t_{DT} for 10 droplets**	3960 s	206 s
**Standard deviation for 10 droplets**	751 s	68 s

Table S3 Thermal conductivity of all samples

Sample	Thermal conductivity (W/m k)
Copper sheet	403
MOF-M_0NS	361.2
MOF-M_{50}NS	333.6
MOF-M_{100}NS	341.7
MOF-M_{150}NS	310.4
MOF-M_{200}NS	286.3
MOF-M_{250}NS	296.4
MOF-M_{300}NS	301.2

Table S4 Parameters of water droplet on surfaces in delayed icing process

Parameters	Values (MOF-M_{250}NSsurface)	Values (Cu sheet)
α (in natural convection conditions)	5 W/m²·K	5 W/m²·k
C_n (W/m²·K)	0.97	Null
λ (W/m·K)	296.4	403
R (m)	1 × 10^{-3}	1.5 × 10^{-3} m
β (°)	159	92
x (m)	5 × 10^{-4}	5× 10^{-4}
T_d (°C)	9	9
T_{sub} (°C)	-15.6	-9.3
T_s (°C)	-18.2	-11.7
t (s)	3960 s	262 s

5. Supplementary Movies

Movie S1: COMOSOL simulation of heat transfer process for MOF-M_{250}NS at -18°C and RH < 20%;

Movie S2: COMOSOL simulation of heat transfer process for flat copper sheet at -18°C and RH < 20%;

Movie S3: Frost formation process of MOF-M_0NS and MOF-M_{250}NS at -20°C and RH ~ 60%;

Movie S4: Defrosting process of MOF-M_{250}NS, (b) MOF-M_0NS and (c) copper sheet at a low temperature of −20 °C and RH ~ 60% under 1-sun illumination;

Movie S5: Deicinging process of MOF-M_{250}NS, (b) MOF-M_0NS and (c) copper sheet at a low temperature of −20 °C and RH ~ 80% under 1-sun illumination;

Movie S6: Deicinging process of MOF-M_{250}NS at a low temperature of −20 °C and RH ~ 80% under 0.7-sun and 0.5-sun illumination;

Movie S7: Low-temperature self-cleaning process of MOF-M_{250}NS, the environmental temperature is set to – 40 °C.
